# Properties of cardiac conduction in a cell-based computational model

**DOI:** 10.1371/journal.pcbi.1007042

**Published:** 2019-05-31

**Authors:** Karoline Horgmo Jæger, Andrew G. Edwards, Andrew McCulloch, Aslak Tveito

**Affiliations:** 1 Simula Research Laboratory, Oslo, Norway; 2 Department of Bioengineering, University of California, San Diego, California, United States of America; Cardiovascular research laboratory, UCLA, UNITED STATES

## Abstract

The conduction of electrical signals through cardiac tissue is essential for maintaining the function of the heart, and conduction abnormalities are known to potentially lead to life-threatening arrhythmias. The properties of cardiac conduction have therefore been the topic of intense study for decades, but a number of questions related to the mechanisms of conduction still remain unresolved. In this paper, we demonstrate how the so-called EMI model may be used to study some of these open questions. In the EMI model, the extracellular space, the cell membrane, the intracellular space and the cell connections are all represented as separate parts of the computational domain, and the model therefore allows for study of local properties that are hard to represent in the classical homogenized bidomain or monodomain models commonly used to study cardiac conduction. We conclude that a non-uniform sodium channel distribution increases the conduction velocity and decreases the time delays over gap junctions of reduced coupling in the EMI model simulations. We also present a theoretical optimal cell length with respect to conduction velocity and consider the possibility of ephaptic coupling (i.e. cell-to-cell coupling through the extracellular potential) acting as an alternative or supporting mechanism to gap junction coupling. We conclude that for a non-uniform distribution of sodium channels and a sufficiently small intercellular distance, ephaptic coupling can influence the dynamics of the sodium channels and potentially provide cell-to-cell coupling when the gap junction connection is absent.

## Author summary

The electrochemical wave traversing the heart during every beat is essential for cardiac pumping function and supply of blood to the body. Understanding the stability of this wave is crucial to understanding how lethal arrhythmias are generated. Despite this importance, our knowledge of the physical determinants of wave propagation are still evolving. One particular challenge has been the lack of accurate mathematical models of conduction at the cellular level. Because cardiac muscle is an electrical syncytium, in which direct charge transfer between cells drives wave propagation, classical bidomain and monodomain tissue models employ a homogenized approximation of this process. This approximation is not valid at the length scale of single cells, and prevents any analysis of how cellular structures impact cardiac conduction. Instead, so-called microdomain models must be used for these questions. Here we utilize a recently developed modelling framework that is well suited to represent small collections of cells. By applying this framework, we show that concentration of sodium channels at the longitudinal borders of myocytes accelerates cardiac conduction. We also demonstrate that when juxtaposed cells are sufficiently close, this non-uniform distribution induces large ephaptic currents, which contribute to intercellular coupling.

## Introduction

The contraction of the heart is initiated by an electrical signal spreading through the cardiac muscle, triggering the cardiomyocytes to contract in synchrony. The conduction of this signal from myocyte to myocyte is therefore essential for maintaining the pumping function of the heart and it is well established that abnormalities in cardiac conduction are associated with an increased risk of life-threatening arrhythmias (see e.g., [1, 2, 3]).

Cardiac conduction was long believed to be continuous in nature, with low resistance gap junctions allowing for a virtually continuous conduction of electrical signals between cells (see e.g., [4]). This view was challenged when experiments in the 1980s revealed that, even though the conduction velocity was faster in the direction along the cardiac fibers than in the transverse direction, the maximal upstroke velocity was higher for transverse propagation than for longitudinal propagation [5, 6]. This observation was not in agreement with the assumption of continuous conduction, because in a continuous medium, the conduction velocity would be expected to directly correspond to the maximal upstroke velocity, in the sense that a fast conduction velocity would be associated with a fast upstroke velocity [7]. The experiments therefore suggested that there might be discontinuities in the intracellular resistivity and it was theorized that these discontinuities might be explained by gap junctions with a considerably higher resistance than previously assumed. Moreover, direct measurements of the gap junction resistance supported this claim and showed that the resistance at the intercalated discs between adjacent cells was approximately the same as the axial resistance of the cell [8, 9]. Today it is considered well established that cardiac conduction is discontinuous [7], and this raises questions of, for example, how the shape and size of the cardiomyocytes affect the conduction velocity and how this is influenced by the distribution of gap junctions (see e.g., [10, 11, 12, 13, 14, 15, 16]).

Another experimental finding challenging the classical views of cardiac conduction was done in 2012, when it was demonstrated that the conduction velocity decreased as the size of the extracellular space in guinea pig ventricular myocardium was increased [17]. This is the opposite of what is expected from mathematical considerations based on classical cable theory (see e.g., [18]), which predicts that the conduction velocity should increase as the size of the extracellular space increases (see e.g., [2, 14]). In addition, the experiments showed that an increased extracellular volume was associated with an increased sensitivity to gap junction uncoupling [17].

These results seem to support the claim that other mechanisms than the gap junctions might serve as alternative or supporting pathways for spreading the electrical signals from cell to cell (see e.g., [19]). In particular, the results seem to support the hypothesis of ephaptic coupling (i.e. coupling through the extracellular space) acting as a supporting mechanism for cardiac conduction. A number of computational studies have supported the viability of this hypothesis (e.g., [20, 21, 22, 23, 24, 25, 26, 27, 28, 29]), although the effect appears to depend highly on certain parameters. Specifically, the distance between the cells must be relatively small and the sodium channels must be highly localized at the intercalated discs in order for the electrical signal to pass between cells through ephaptic coupling alone. Sodium channels have in fact been demonstrated to highly localize at the intercalated discs (see e.g., [30, 31, 32 22, 28, 17]) and the precise consequences of such a non-uniform distribution of sodium channels is another open question (see e.g., [20, 22, 33]).

The understanding of cardiac conduction has evolved by both experimental measurements and by theoretical considerations using mathematical models. The mathematical bidomain and monodomain models, for instance, have been used extensively to study propagating waves in cardiac tissue [34], and the models have been incorporated into several major software projects like Chaste, Carp, Continuity, and FEniCS [35, 36, 37, 38]. The bidomain and monodomain models allow for directional intracellular and extracellular resistivities accounting for the anisotropic nature of cardiac conduction. However, the models represent the cardiac tissue in a homogenized manner, and the intracellular space, the extracellular space and the cell membrane are all assumed to exist everywhere in the tissue. Moreover, the resistance of the gap junctions is generally incorporated into the intracellular resistivity in an averaged manner [4]. Consequently, these models might not be sufficient for representing mechanisms related to the discontinuous nature of cardiac conduction. Also, as pointed out in [39], a non-uniform distribution of ion channels is difficult to represent realistically in homogenized models.

In order to study these mechanisms, several alternative models have been introduced, representing the discrete nature of cardiac tissue with different levels of detail and complexity (see e.g., [40, 41, 22, 25, 26, 27, 23]). In this paper, we consider a detailed model which has been applied in several earlier studies of excitable cells, including studies of collections of cardiomyocytes (e.g., [42, 43, 44, 45, 46, 47, 48, 49, 50, 51, 39]). We refer to the model as the EMI model because it includes an explicit representation of the **E**xtracellular space, the cell **M**embrane and the **I**ntracellular space. In addition, the intercalated discs between adjacent cells are incorporated into the model as membranes with capacitive and resistive properties. The model thus allows for a more detailed analysis of the properties of cardiac conduction than the classical bidomain and monodomain models. For example, the possibility to represent the cell connections explicitly allows for investigations of the discontinuous nature of conduction. Similarly, the explicit representation of the extracellular space makes the model applicable for studying the ephaptic coupling between cells and the effect of the size of the extracellular space on the conduction velocity. Furthermore, the EMI model is well-suited for studying non-uniform distributions of ion channels on the cell membrane because the geometry of each cell is explicitly defined in the model.

In our computations, we show that when the sodium channels are located at the cell ends, the conduction velocity increases and the time delays across gap junctions shorten compared to the case of a uniform sodium channel distribution. We also observe that there are large changes in the extracellular potential in the clefts between cells during propagation, leading to changes in the sodium channel dynamics and potentially enabling cell coupling through ephaptic coupling.

## Methods

In this section, we define the EMI model used in our investigations, as well as the models chosen for the membrane and gap junction dynamics. In addition, we describe the cell geometry and the numerical methods used in our computations.

### The EMI model

The EMI model will be used to simulate small collections of cardiomyocytes. For simplicity, we here describe the EMI model in the case of just two coupled cells. The extension to larger collections of cells follows directly from the two-cell definition.

**Fig 1:**
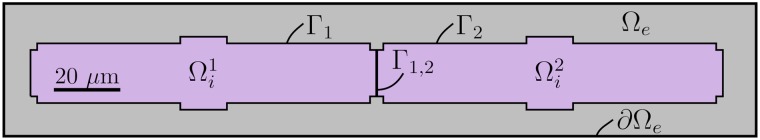
Illustration of a 2D version of the computational domain for two cells. The domain consists of an extracellular domain Ω_*e*_ and two intracellular domains $\Omega_{i}^{1}$ and $\Omega_{i}^{2}$ with cell membranes Γ_1_ and Γ_2_. The intracellular domains are connected by the intercalated disc denoted by Γ_1,2_, and the outer boundary of the extracellular domain is denoted by ∂Ω_*e*_.

A two-dimensional (2D) version of the components of the EMI model for two connected cells is illustrated in Fig 1. Note, however, that in all our simulations, we consider a three-dimensional (3D) version of the model. The domain consists of two intracellular domains $\Omega_{i}^{1}$ and $\Omega_{i}^{2}$ surrounded by an extracellular domain Ω_*e*_. On the interface between the extracellular domain and the intracellular domain $\Omega_{i}^{k}$, we define a cell membrane denoted by Γ_*k*_ for *k* = 1, 2. Similarly, on the interface between the two intracellular domains $\Omega_{i}^{1}$ and $\Omega_{i}^{2}$, we define an intercalated disc Γ_1,2_. The outer boundary of the extracellular domain is denoted by ∂Ω_*e*_, and we separate this boundary into two parts, $\partial \Omega_{e}=\partial \Omega_{e}^{D}\;\cup \;\partial \Omega_{e}^{N}$, where a Dirichlet boundary condition is applied on $\Omega_{e}^{D}$ and a Neumann boundary condition is applied on $\Omega_{e}^{N}$.

The EMI model describes the electrical potential in these domains and is described in detail in [39], however, for completeness, we repeat the model formulation here. For two connected cells, the EMI model reads

$$\nabla \cdot \sigma_{e}\nabla u_{e}=0\;\;\text{in}\;\Omega_{e},$$ (1)

$$\nabla \cdot \sigma_{i}\nabla u_{i}^{k}=0\;\;\text{in}\;\Omega_{i}^{k},$$ (2)

$$u_{e}=0\;\;\text{at}\;\partial \Omega_{e}^{D},$$ (3)

$$\sigma_{e}\frac{\partial u_{e}}{\partial n_{e}}=0\;\;\text{at}\;\partial \Omega_{e}^{N},$$ (4)

$$n_{e}\cdot \sigma_{e}\nabla u_{e}=-n_{i}^{k}\cdot \sigma_{i}\nabla u_{i}^{k}\equiv I_{m}^{k}\;\;\text{at}\;\Gamma_{k},$$ (5)

$$u_{i}^{k}-u_{e}=v^{k}\;\;\text{at}\;\Gamma_{k},$$ (6)

$$v_{t}^{k}=\frac{1}{C_{m}}\left(I_{m}^{k}-I_{\text{ion}}^{k}\right)\;\;\text{at}\;\Gamma_{k},$$ (7)

$$n_{i}^{2}\cdot \sigma_{i}\nabla u_{i}^{2}=-n_{i}^{1}\cdot \sigma_{i}\nabla u_{i}^{1}\equiv I_{1,2}\;\;\text{at}\;\Gamma_{1,2},$$ (8)

$$u_{i}^{1}-u_{i}^{2}=w\;\;\text{at}\;\Gamma_{1,2},$$ (9)

$$w_{t}=\frac{1}{C_{1,2}}\left(I_{1,2}-I_{\text{gap}}\right)\;\;\text{at}\;\Gamma_{1,2},$$ (10)

for *k* = 1, 2. Here, the variables of the model are the extracellular potential *u*_*e*_ defined in Ω_*e*_, the intracellular potentials $u_{i}^{1}$ and $u_{i}^{2}$ defined in $\Omega_{i}^{1}$ and $\Omega_{i}^{2}$, respectively, the membrane potentials *v*^1^ and *v*^2^ defined on the membranes Γ^1^ and Γ^2^, respectively, and *w* defined on the intercalated disc, Γ_1,2_. All potentials are given in mV. Furthermore, *σ*_*i*_ and *σ*_*e*_ are the intracellular and extracellular conductivities, respectively, given in mS/cm, and *n*_*e*_ and $n_{i}^{k}$, are the outward pointing normal vectors of Ω_*e*_ and $\Omega_{i}^{k}$, respectively. The current density $I_{\text{ion}}^{k}$ represents the ionic currents across the membrane and *I*_gap_ represents the ionic current density through the gap junctions. Similarly, $I_{m}^{k}$ represents the sum of the ionic and capacitive current densities across the membrane, and *I*_1,2_ represents the sum of the ionic current density through the gap junctions and the capacitive current density of the intercalated disc. All the current densities $I_{\text{ion}}^{k}$, *I*_gap_, $I_{m}^{k}$ and *I*_1,2_ are given in *μ*A/cm^2^. The parameters *C*_*m*_ and *C*_1,2_ represent the specific capacitance of the cell membrane and the intercalated disc, respectively and are given in *μ*F/cm^2^. Moreover, time is given in ms and length is given in cm.

In our simulations, we apply the Dirichlet boundary condition (3) on the outer boundary of the extracellular domain in the *x*- and *y*-directions and the Neumann boundary condition (4) in the *z*-direction. The default parameter values used in the simulations are given in Table 1.

**Table 1:** Default parameter values used in the simulations. For the parameters used in the Grandi et al. model, we refer to [55].

Parameter	Value	Ref.
*C*_*m*_	1 *μ*F/cm^2^	[52]
*C*_1,2_	0.5 *μ*F/cm^2^	
*σ*_*i*_	4 mS/cm	[53, 54]
*σ*_*e*_	20 mS/cm	[50]
*R*_*g*_	0.0045 kΩcm^2^	
Size Ω_O_	100 *μ*m × 18 *μ*m × 18 *μ*m	
Size Ω_W_, Ω_E_	2 *μ*m × 14 *μ*m × 14 *μ*m	
Size Ω_S_, Ω_N_	14 *μ*m × 2 *μ*m × 14 *μ*m	
Δ*x*, Δ*y*	1 *μ*m	
Δ*z*	2 *μ*m	
Δ*t* (PDE part)	0.001 ms	
Δt* (ODE part)	min(0.001 ms, Δ*t*)	

### Membrane dynamics

The ionic current density $I_{\text{ion}}^{k}$ across the membrane between the intracellular and extracellular domains represents the sum of a large number of currents through various ion channels, pumps and exchangers located on the cell membrane. We use the epicardial version of the Grandi et al. action potential model [55] to model these currents. This model includes several state variables in addition to the membrane potential *v*, representing, for instance, intracellular ionic concentrations and gating variables of the ion channels. In order to account for these additional state variables, the EMI model given by (1)–(10) is extended to include a system of ordinary differential equations of the form

$$s_{t}=F\left(v,s\right)$$ (11)

for the dynamics of the state variables. The equations defining the right-hand side *F*(*v*, *s*) is here given directly by the Grandi model. All state variables are defined only on the cell membrane and the value of the state variables is allowed to vary both in time and space. In other words, each state variable may have different values at different locations on the cell membrane.

In our simulations, we use the default initial conditions of the Grandi model for all the state variables governing the membrane dynamics, including the membrane potential *v*. Unless otherwise stated, we initiate the propagating wave in the simulations by stimulating the first two cells in the *x*-direction by a 1 ms long stimulus current of 80 A/F.

### Gap junctions

The ionic currents through the gap junctions are modelled by the passive model

$$I_{\text{gap}}=\frac{1}{R_{g}}w.$$ (12)

Here, *R*_*g*_ represents the resistance of the gap junctions (in kΩcm^2^), and $G_{g}=\frac{1}{R_{g}}$ (in mS/cm^2^) is the conductance of the gap junctions. We use the initial condition *w* = 0 mV for the gap junction potential.

### Numerical method

The EMI system (1)–(11) is solved using an operator splitting procedure. This numerical scheme is described in detail in [39]. In short, for every time step *t*_*n*_ = *n*Δ*t*, the system (1)–(11) is solved in two steps. First, the system (11) and (7) with $I_{m}^{k}$ set to zero is solved by *m* forward Euler steps of size Δt* = Δ*t*/*m*. Then, in the second step of the operator splitting procedure, the system (1)–(10) with $I_{\text{ion}}^{k}$ set to zero is solved by a single step of an implicit finite difference discretization of the linear system.

The default discretization parameters used in the simulations are given in Table 1. Note that for simulations with time steps, Δ*t*, larger than 0.001 ms, the time step Δt* = 0.001 ms is still used for the ODE step of the operator splitting procedure, but when values of Δ*t* < 0.001 ms is used, Δt* is set equal to this value of Δ*t* < 0.001 ms.

### Domain and cell geometry

**Fig 2:**
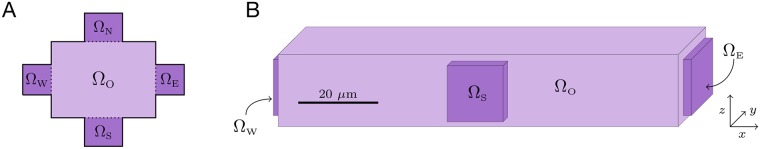
Cell geometry used in the simulations. A: Illustration of a two-dimensional version of a single cell. The intracellular domain is composed of the subdomains Ω_O_, Ω_W_, Ω_E_, Ω_S_, and Ω_N_. B: Three-dimensional illustration of the geometry of a single cell based on the default cell size described in Table 1.

In our computations, we consider a 3D domain consisting of an extracellular space and a single strand of 3D cells. The cells are connected to each other in a single row in the *x*-direction by gap junctions. Because simple, rectangular geometries are most conveniently handled by the finite difference scheme used in the computations, we consider a very simplified cell geometry. Two- and three-dimensional illustrations of the cell shape used in the simulations are given in the left and right panels of Fig 2, respectively. Each cell is a composition of the intracellular domains Ω_O_, Ω_W_, Ω_E_, Ω_S_, and Ω_N_, and each of these domains is shaped as a rectangular cuboid. The cells may connect to each other by gap junctions like illustrated for two cells in Fig 1. The default cell sizes used in our computations are given in Table 1. The minimal distance between the intracellular domain and the outer boundary of the extracellular domain is 10 *μ*m in the *x*- and *y*-directions and 4 *μ*m in the *z*-direction.

### Model parameters

The default parameter values used in the simulation are given in Table 1. The values for the specific membrane capacitance, *C*_*m*_, and the intracellular and extracellular conductivities, *σ*_*i*_ and *σ*_*e*_, are taken from literature (see [52, 53, 54, 50]). The value of *C*_1,2_ is set to *C*_*m*_/2 because the intercalated disc is assumed to be a membrane of thickness twice as large as the cell membrane, and the specific capacitance of a capacitor formed by two parallel plates separated by an insulator may be assumed to be inversely proportional to the thickness of the insulator [56]. The value for *R*_*g*_ was chosen so that the conduction velocity for the uniform distribution of sodium channels (see the next section) was approximately 50 cm/s (in rough agreement with e.g., [57, 58]).

### Distribution of sodium channels

In order to study how the distribution of sodium channels on the cell membrane affects the properties of conduction, we consider both a uniform sodium channel distribution (U) and a non-uniform distribution (NU). In the uniform case, the sodium channels are distributed evenly over the entire membrane. This means that the sodium channel conductance is the same in all computational nodes located on the cell membrane, and this value is set equal to the default value in the Grandi et al. action potential model [55].

**Fig 3:**
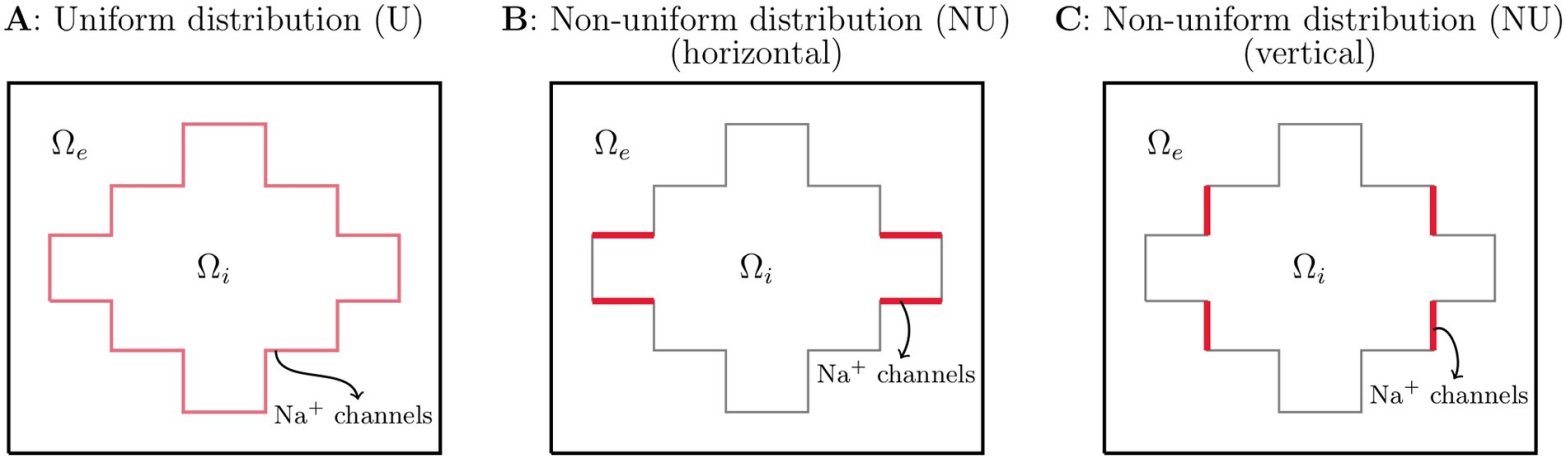
Illustration of three different spatial distributions of the sodium channels for a 2D single cell case. A: Uniform distribution. B: Non-uniform distribution with a high density of sodium channels at the horizontal part of the cell ends in the *x*-direction. C: Non-uniform distribution with a high density of sodium channels on the vertical part of the cell ends in the *x*-direction.

In the non-uniform case, some or all of the sodium channels are moved to an area close to the cell ends in the *x*-direction. For most of the simulations, this area is the membrane of Ω_W_, Ω_E_ (see Fig 3B). However, for the simulations investigating ephaptic coupling, we place the sodium channels on the vertical part of the cell membrane between two cells (see Fig 3C). This is done in order to keep the size of areas with sodium channels constant for different cell distances and because we in these simulations consider much smaller cell distances than in the remaining simulations. In the remaining simulations, the distance between the cells is quite large (4 *μ*m), and the sodium channels are placed on the horizontal part of the cell ends in order to locate the channels as close to the cell connections as possible.

When studying how the conduction properties are affected by different sodium channel distributions, we let the total sodium channel conductance of each cell be the same for the different sodium channel distributions. We define this total sodium channel conductance as

$$G_{\text{Na}}=\int_{\Gamma }g_{\text{Na}}dS,$$ (13)

where *g*_Na_ is the sodium channel conductance density (in mS/cm^2^) and Γ is the cell membrane. If ${\bar{g}}_{\text{Na}}$ is the default value of the sodium channel conductance density used in the uniform case and *A*_*c*_ is the total membrane area of the cell, the total sodium channel conductance in the uniform case is

$$G_{\text{Na},U}=\int_{\Gamma }{\bar{g}}_{\text{Na}}dS=A_{c}{\bar{g}}_{\text{Na}}.$$ (14)

In the non-uniform case, we divide the membrane into a part with an increased sodium channel density, Γ_*j*_, and the remaining part, Γ_*r*_ = Γ\Γ_*j*_. On Γ_*r*_, we let the sodium channel conductance density be given by

$$g_{\text{Na},r}=\left(1-p\right){\bar{g}}_{\text{Na}},$$ (15)

where *p* is the fraction of sodium channels moved to the cell ends. On Γ_*j*_, we let the value be given by

$$g_{\text{Na},j}=g_{\text{Na},r}+p\frac{A_{c}}{A_{j}}{\bar{g}}_{\text{Na}},$$ (16)

where *A*_*j*_ is the area of Γ_*j*_. This way, the total sodium channel conductance in the non-uniform case is

$$G_{\text{Na},\text{NU}}=\int_{\Gamma_{r}}g_{\text{Na},r}dS+\int_{\Gamma_{j}}g_{\text{Na},j}dS$$ (17)

$$=\left(A_{c}-A_{j}\right)g_{\text{Na},r}+A_{j}(g_{\text{Na},r}+p\frac{A_{c}}{A_{j}}{\bar{g}}_{\text{Na}})$$ (18)

$$=\left(1-p\right)A_{c}{\bar{g}}_{\text{Na}}+pA_{c}{\bar{g}}_{\text{Na}}=A_{c}{\bar{g}}_{\text{Na}}$$ (19)

$$=G_{\text{Na},\text{U}}\cdot$$ (20)

In the case when all the sodium channels are located at the cell ends(i.e., *p* = 1), this means that

$$g_{\text{Na},r}=0,$$ (21)

$$g_{\text{Na},j}=\frac{A_{c}}{A_{j}}{\bar{g}}_{\text{Na}}.$$ (22)

### Numerical representation of integrals and areas

The areas *A*_*c*_ and *A*_*j*_ used in the definition (16) are computed by numerical integration over the computational nodes representing the membrane, and the junctional part of the membrane, respectively. More specifically, the areas are computed by

$$A_{c}=\int_{\Gamma }1dS\approx \sum_{i\in I_{\Gamma }}A_{i},$$ (23)

$$A_{j}=\int_{\Gamma_{j}}1dS\approx \sum_{i\in I_{\Gamma_{j}}}A_{i},$$ (24)

where *I*_Γ_ and $I_{\Gamma_{j}}$ denote the collection of the indices of all computational nodes located on the membrane and the junctional part of the membrane, respectively. Furthermore *A*_*i*_ are areas associated with each of the computational membrane nodes *i*. These areas are defined as

$$\begin{array}{c} A_{i}=\{\begin{array}{cc} \Delta x\Delta y, & \text{for}\;\text{nodes}\;\text{on}\;\text{a}\;\text{membrane}\;\text{plane}\;\text{that}\;\text{is}\;\text{constant}\;\text{in}\;\text{the}\;z\text{-direction,}\\ \Delta x\Delta z, & \text{for}\;\text{nodes}\;\text{on}\;\text{a}\;\text{membrane}\;\text{plane}\;\text{that}\;\text{is}\;\text{constant}\;\text{in}\;\text{the}\;y\text{-direction,}\\ \Delta y\Delta z, & \text{for}\;\text{nodes}\;\text{on}\;\text{a}\;\text{membrane}\;\text{plane}\;\text{that}\;\text{is}\;\text{constant}\;\text{in}\;\text{the}\;x\text{-direction,}\\ \frac{1}{2}\left(\Delta x\Delta y+\Delta x\Delta z\right), & \text{for}\;\text{nodes}\;\text{at}\;\text{the}\;\text{intersection}\;\text{of}\;\text{membrane}\;\text{planes}\;\text{constant}\;\text{in}\;\text{the}\;z-\;\text{and}\;y\text{-directions,}\\ \frac{1}{2}\left(\Delta x\Delta y+\Delta y\Delta z\right), & \text{for}\;\text{nodes}\;\text{at}\;\text{the}\;\text{intersection}\;\text{of}\;\text{membrane}\;\text{planes}\;\text{constant}\;\text{in}\;\text{the}\;z-\;\text{and}\;x\text{-directions,}\\ \frac{1}{2}\left(\Delta x\Delta z+\Delta y\Delta z\right), & \text{for}\;\text{nodes}\;\text{at}\;\text{the}\;\text{intersection}\;\text{of}\;\text{membrane}\;\text{planes}\;\text{constant}\;\text{in}\;\text{the}\;x-\;\text{and}\;y\text{-directions,}\\ \frac{1}{3}\left(\Delta x\Delta y+\Delta x\Delta z+\Delta y\Delta z\right), & \text{for}\;\text{nodes}\;\text{at}\;\text{the}\;\text{membrane}\;\text{corners.} \end{array} \end{array}$$

## Results

In this section, we demonstrate how the EMI model may be used to investigate properties of cardiac conduction. First, we consider how a non-uniform distribution of sodium channels affects the conduction velocity, the discontinuous nature of conduction and the time delays across gap junctions of reduced coupling. We also consider how the conduction velocity along a strand of cells is affected by the length of the cells. Finally, we use the EMI model to study the possibility of ephaptic coupling acting as an alternative pathway for conduction between cells and investigate how the sodium channel dynamics are affected by ephaptic effects.

### Effect of sodium channel distribution on conduction velocity

**Fig 4:**
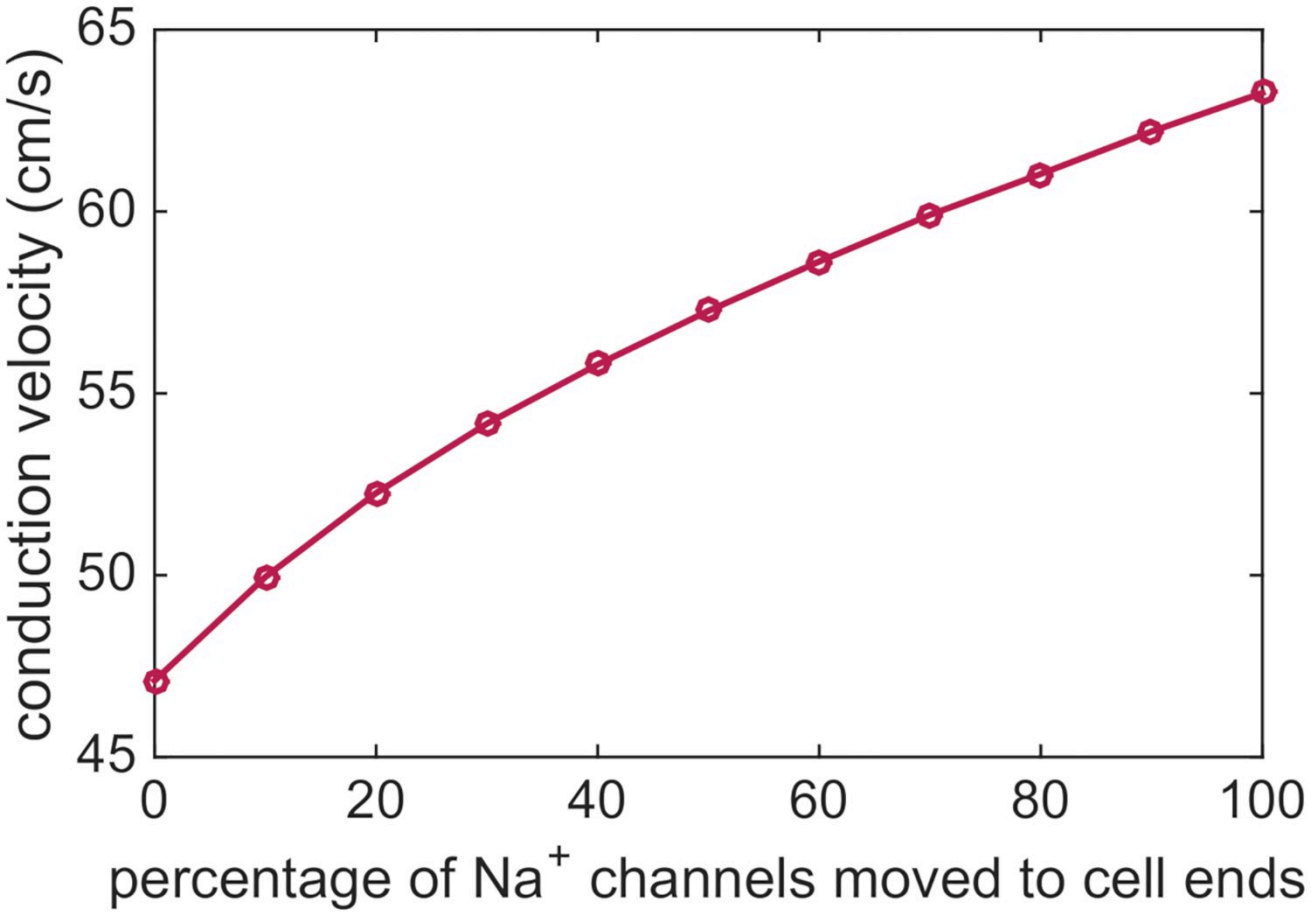
The conduction velocity increases as a larger percentage of the sodium channels is moved to the cell ends. The parameters used in the simulations are given in Table 1, and the simulation includes 15 cells. The conduction velocity is computed as the distance between the cell centers of the fifth and tenth cells divided by the time between the two cell centers first reach a membrane potential of *v* = 0 mV.

As a first example of the application of the EMI model, we will use the model to study how a non-uniform distribution of sodium channels on the cell membrane affects the conduction velocity. In Fig 4, we show the conduction velocity computed in a number of simulations of a strand of 15 cells with an increasing percentages of sodium channels moved to the horizontal parts of the cell ends (see Fig 3B). Here, 0% represents the uniform case, and 100% represents the non-uniform case, when all sodium channels are located close to the cell ends. The total sodium channel conductance of the cell remains the same in each simulation as explained above. We observe that as a larger percentage of the sodium channels are moved to the cell ends, the conduction velocity increases.

Since the largest difference from the uniform case is observed for the case when all sodium channels are moved to the cell ends, we will in the experiments below compare just these two extremes; the uniform case with a constant distribution of sodium channels on the entire membrane and the non-uniform case with all sodium channels located near the cell ends.

### Discontinuous conduction

**Fig 5:**
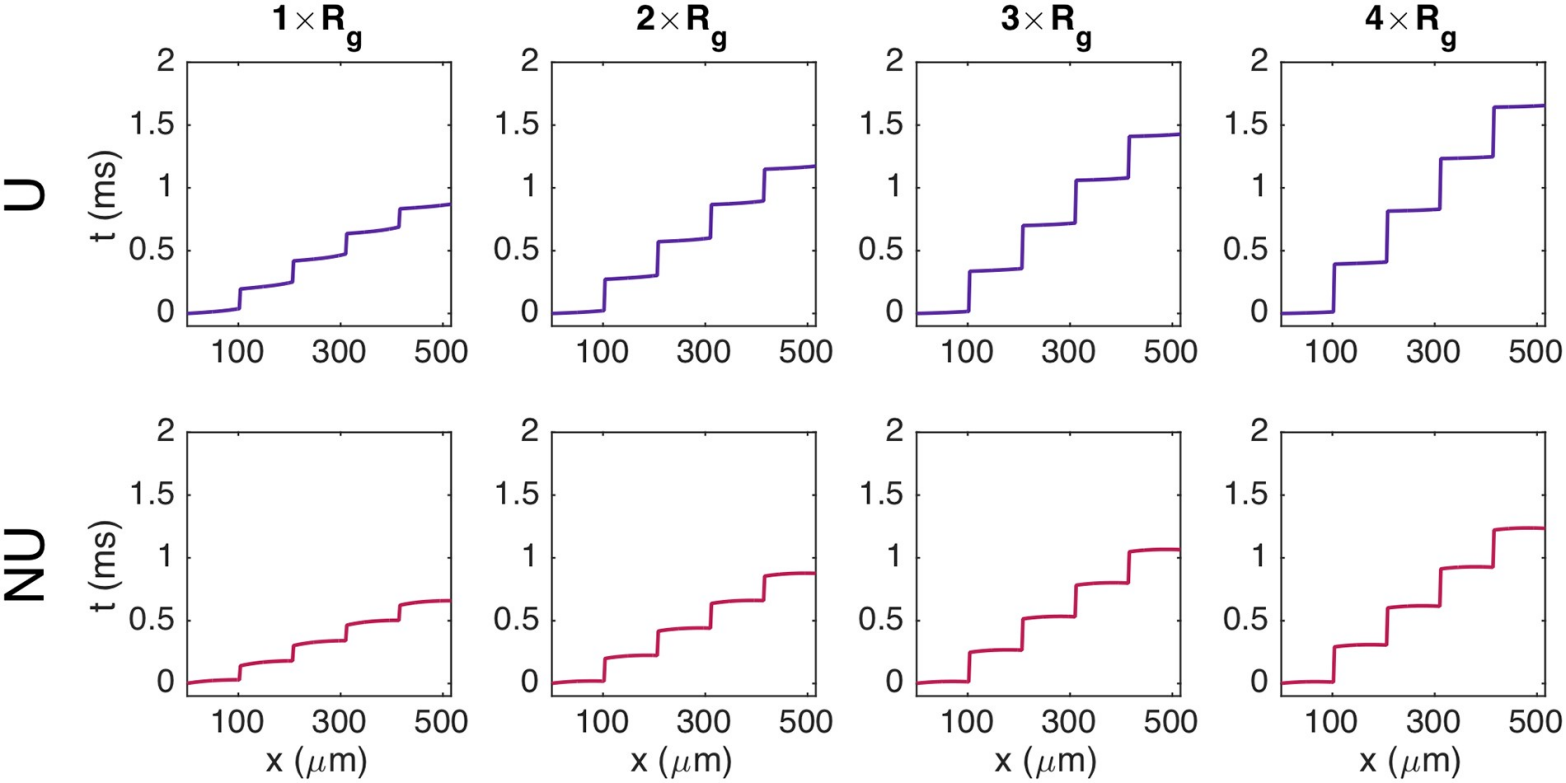
Illustration of discontinuous conduction for a uniform (U) and a non-uniform (NU) distribution of sodium channels. The plots show the time at which each of the *x*-values along the membrane of the five center cells in a simulation of a strand of seven cells first reach a membrane potential of *v* = 0 mV. The title above each panel indicates the factor with which the default value of *R*_*g*_ = 0.0045 kΩcm^2^ is multiplied in the simulation. The remaining parameter values are specified in Table 1, except that the time step is set to Δ*t* = 0.0005 ms.

It is considered well-established that the electrical conduction in cardiac tissue is discontinuous with significant conduction delays when the wave crosses the gap junctions [7]. This discontinuous conduction is conveniently studied using the EMI model because the boundaries between cells are explicitly represented in the model. In Fig 5, we consider a single strand of cells and show the points in time when each of the *x*-values along the cell membranes first reach a membrane potential of *v* = 0 mV. We consider both a uniform and a non-uniform distribution of the sodium channels (see Fig 3A and 3B), and we consider the case of the default value of *R*_*g*_ in addition to three cases of increased *R*_*g*_. In the figure, we observe that there are clearly visible gaps in time between each part of the gap junctions reach *v* = 0 mV, and that the size of these gaps increases as the gap junction resistance is increased. In addition, we observe that the gaps in time seem to be longer for the uniform case compared to the non-uniform case, and the overall time spent crossing the five cells is longer for the uniform case for all values of *R*_*g*_.

#### Activation times for a single cell

**Fig 6:**
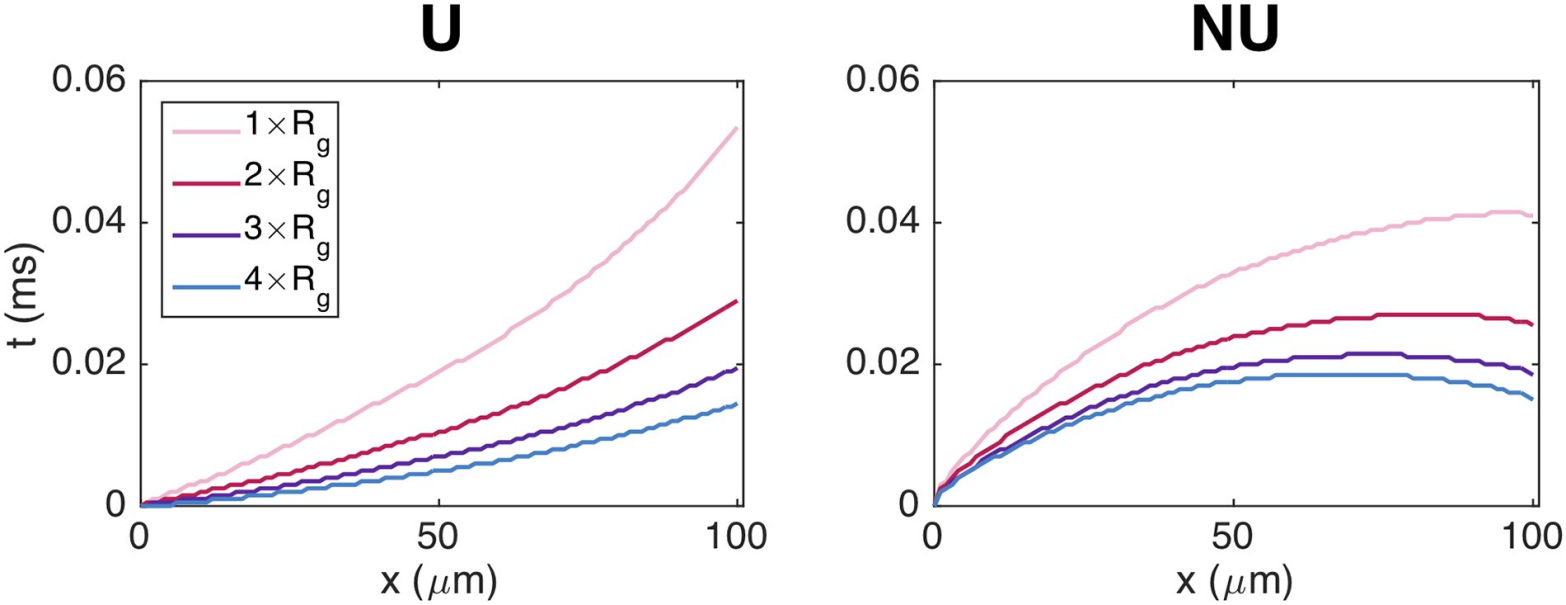
The intracellular activation times are affected by the sodium channel distribution and gap junction resistance. The figure shows the results from Fig 5 zoomed in on the *x*-values corresponding to a single cell in the center of the domain.

In Fig 6, we focus on the *x*-values corresponding to a single cell. We observe that the curves for the activation times are not straight lines, but bend along the length of the cell. Moreover, the shape of the curves is clearly different in the uniform and non-uniform cases. In the uniform case, the curves seem to steepen towards the cell end, while for the non-uniform case, the curves seem to flatten out towards the end of the cell. In fact, for the non-uniform case, the activation time is shorter for the far-right part of the cell than at about 80% of the cell length in the cases of increased gap junction resistance.

Furthermore, we observe that for both sodium channel distributions, the time between the start and the end of the cell reaches *v* = 0 mV is shorter for the case with a high gap junction resistance compared to the case with the default value. This means that wave travels faster over a single cell as the gap junction resistance is increased and, as seen in Fig 5, the time delays across the gap junctions are increased.

### Effect of sodium channel distribution on the upstroke velocity

**Fig 7:**
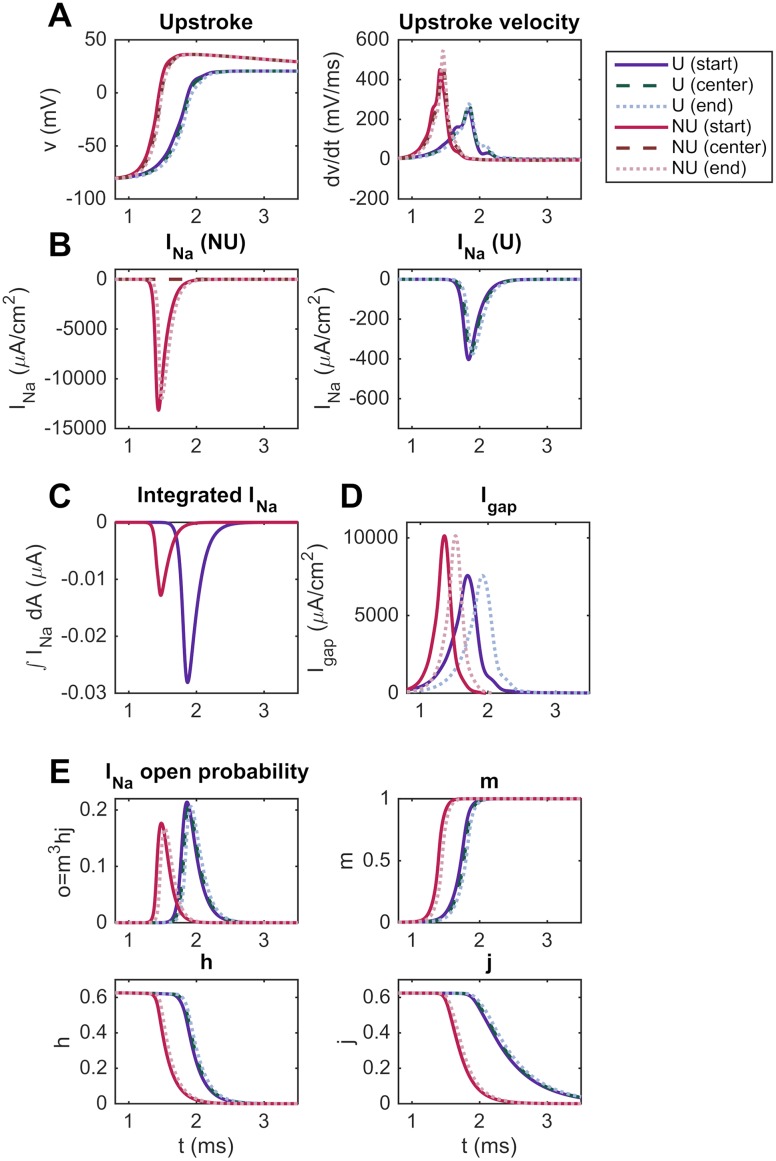
The upstroke velocity and gap junction currents are increased for the NU sodium channel distribution. Comparison of the action potential upstroke and upstroke velocity (A), *I*_Na_ (B), integrated *I*_Na_ (C), *I*_gap_ (D), and gating dynamics of *I*_Na_ (E) for the U and NU cases (see Fig 3A and 3B). The parameters used in the simulations are given in Table 1, and the simulations include 15 cells. We consider the seventh cell and record the membrane potential, upstroke velocity and currents for the first *x*-value, the center *x*-value and the last *x*-value of the cell.

As seen in Fig 4, the conduction velocity is increased for a non-uniform distribution of sodium channels compared to a uniform distribution. To investigate the reason for this effect, we consider the upstroke velocity of the action potential computed for the two sodium channel distributions. In the left panel of Fig 7A, we report how the membrane potential changes with time for a grid point located at the beginning of the seventh cell, at the center of the cell and at the end of the cell in the uniform case and in the non-uniform case with all sodium channels located at the cell ends. In the right panel, we plot the corresponding upstroke velocity (*dv*/*dt*) as a function of time. We observe that the upstroke velocity is quite similar in the three points along the cell, but that the upstroke velocity is clearly increased in the non-uniform case compared to the uniform case. This increased upstroke velocity might explain the increased conduction velocity reported in Fig 4.

#### Effect of the sodium channel distribution on the sodium channel dynamics

In order to investigate the difference in the upstroke velocity observed between the NU and U cases in Fig 7A, we in Fig 7B report the sodium current density at the same three membrane points for the two sodium channel distributions. In the NU case, the sodium current density is zero in the center of the domain, but has a much larger magnitude than for the U case at the points close to the cell ends.

The total sodium current integrated over the entire membrane is reported in Fig 7C, and we observe that the integrated current is larger for the U case than for the NU case. In addition, in Fig 7E, we report the gating dynamics of the sodium current, and we observe that the open probability of the sodium channels is slightly larger for the U case compared to the NU case. The increased upstroke velocity for the NU case therefore does not seem to be caused by a larger total sodium current over the cell membrane, but rather by the locally increased sodium current density close to the cell ends.

#### Effect of the sodium channel distribution on the gap junction current

In Fig 7D, we report the size of the current density through the gap junctions, *I*_gap_, for the NU and U cases. We observe that the gap junction current density is larger for the NU case than for the U case.

**Fig 8:**
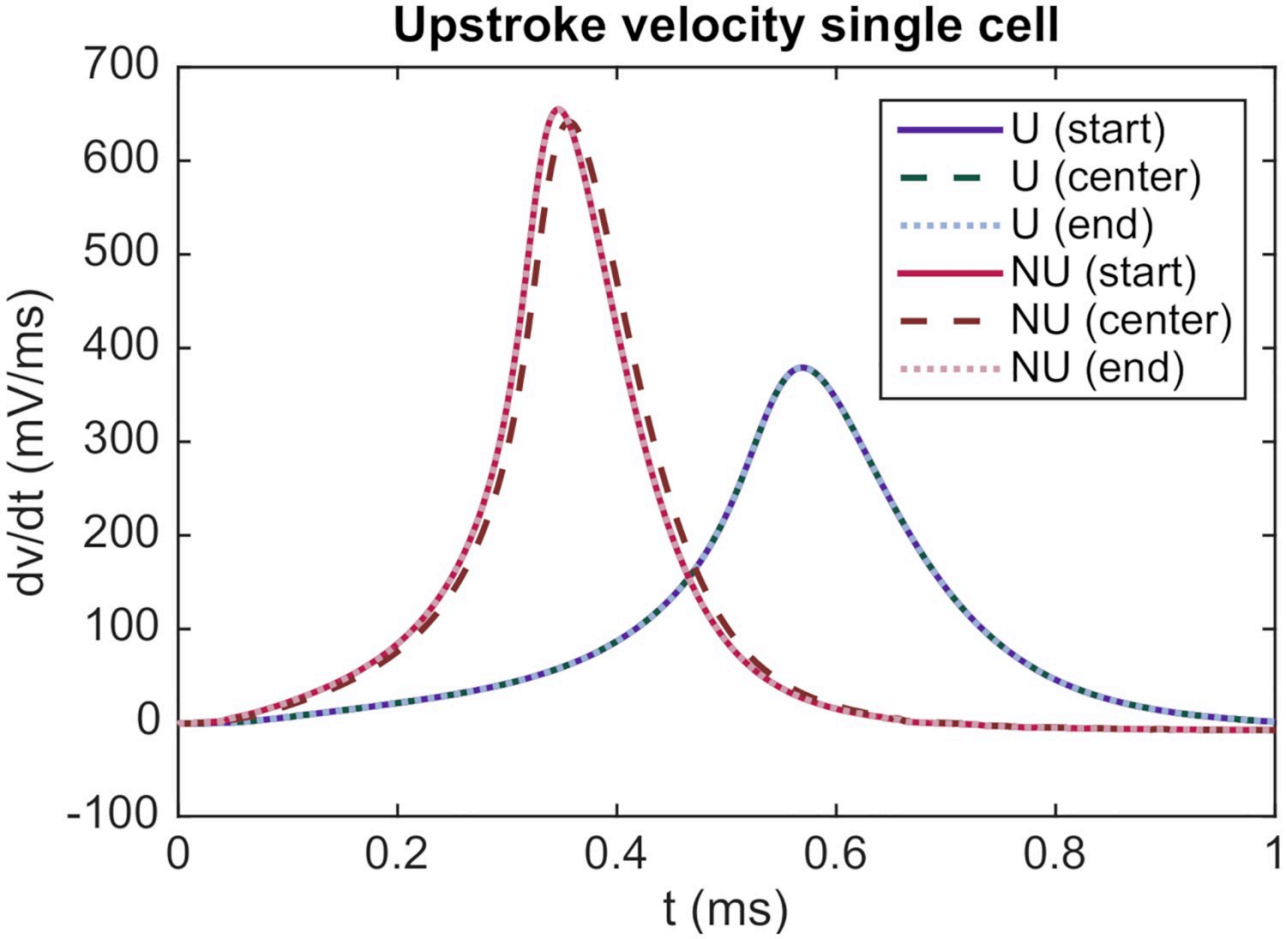
The upstroke velocity is larger for the NU case than for the U case also in a single cell simulation. The plot shows the upstroke velocity at the start of the cell, the center of the cell and the end of the cell for the NU and U sodium channel distributions for a simulation including only a single cell. The cell is triggered by applying the initial condition *v* = −55 mV for all membrane nodes in both the NU and U cases. The parameter values used in the simulation are given in Table 1.

In order to investigate whether the difference in upstroke velocity between the NU and U cases may be explained by this increased *I*_gap_, we report in Fig 8 the upstroke velocity computed for the NU and U cases in a single cell simulation (with no gap junctions present). Again, we observe that the upstroke velocity is considerably higher for the NU case than for the U case. Consequently, the increased upstroke velocity for the NU case observed in Fig 7A seems to be a direct consequence of the locally increased sodium current density at the cell ends and not caused by the increased gap junction currents observed in Fig 7D.

#### Effect of the sodium channel distribution on the time delays across gap junctions

**Fig 9:**
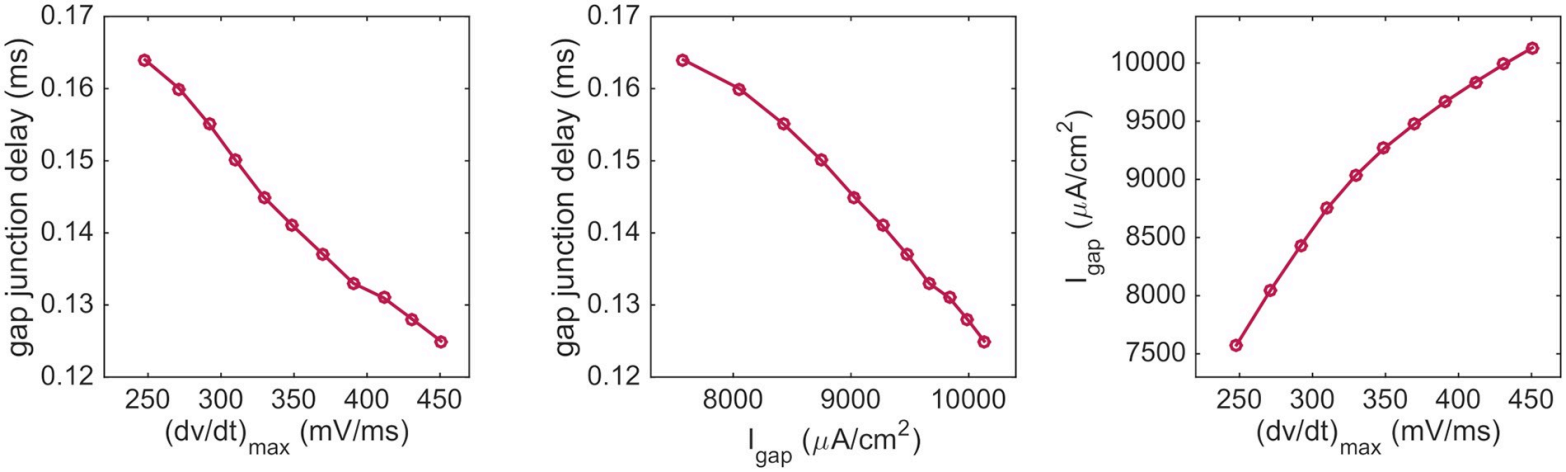
Relationship between the gap junction time delay, maximal upstroke velocity and gap junction current as the percentage of sodium channels moved to the cell ends is changed. The time delay is defined as the time between the membrane potential at the last computational node before the gap junctions between the sixth and seventh cells, and the first computational node after the gap junctions reach 0 mV. The upstroke velocity is computed at the first computational node after the gap junctions. The gap junction current density is computed between the sixth and seventh cell at the center of the domain in the *y*- and *z*-directions. The results are computed for the simulations displayed in Fig 4.

In Figs 5 and 6, we observed that both the gap junction delays and the time spent traversing a single cell was decreased for a non-uniform distribution of sodium channels compared to a uniform distribution. Furthermore, we observed that the travelling wave spends the majority of time crossing the gap junctions. Therefore, decreased gap junction delays could be expected to have the most significant effects on the observed difference in conduction velocity between the NU and U cases. In Fig 9 we report the relationship between the time delays across a gap junction, the maximal upstroke velocity, and the maximal gap junction current density for the simulations reported in Fig 4. We observe that an increased maximal upstroke velocity is associated with increased maximum gap junction currents and decreased time delays across the gap junctions.

### Time delays across gap junctions of reduced coupling

**Fig 10:**
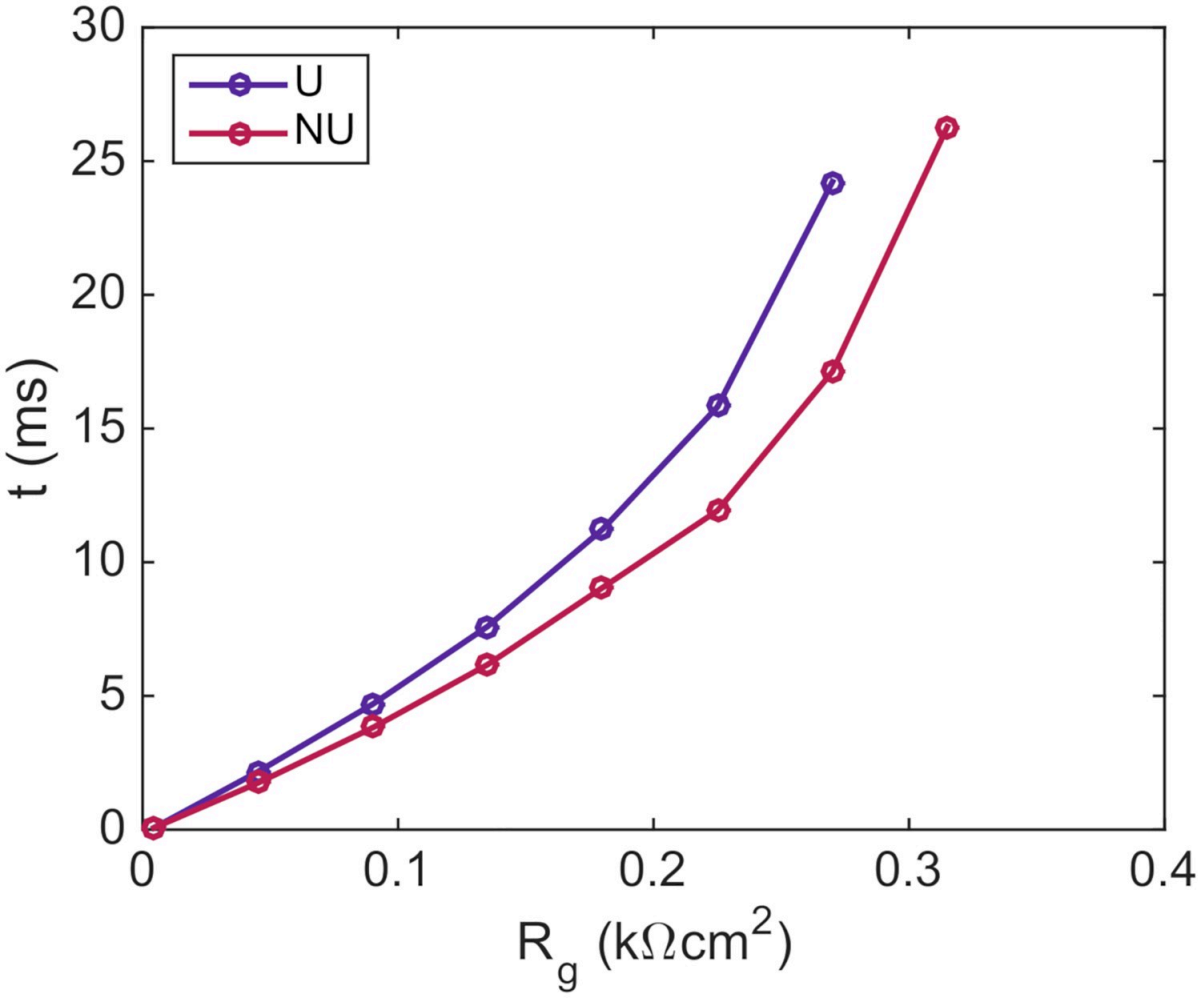
The time delay across gap junctions of reduced coupling is longer for a uniform (U) distribution than for a non-uniform (NU) distribution of sodium channels. The simulation includes a row of ten cells and the gap junction resistance between the fifth and sixth cells is increased by a factor of up to 70 from the default value of *R*_*g*_ = 0.0045 kΩcm^2^. The default value of *R*_*g*_ is used for the remaining gap junctions. The remaining parameters used in the simulations are given in Table 1, except that the time step is set to Δ*t* = 0.01 ms. The timings reported in the plot are the time between the end of the fifth cell and the start of the sixth cell reach a membrane potential of *v* = 0 mV. In the NU case, all the sodium channels are located on the horizontal part of the cell ends (see Fig 3B).

In Fig 10, we show how the time delay over the gap junctions increases as the resistance of the gap junctions are increased. We consider both the case of a uniform distribution of sodium channels and the case of a non-uniform distribution with all sodium channels located close to the cell ends. We observe that the time delays across the gap junctions are longer for the uniform case than for the non-uniform case for all values of the gap junction resistance, *R*_*g*_. Furthermore, the difference between time delays associated with each of the two sodium channel distributions increases as the gap junction resistance is increased. In addition, the value of *R*_*g*_ for which the wave is completely blocked is lower for the uniform case than for the non-uniform case.

**Fig 11:**
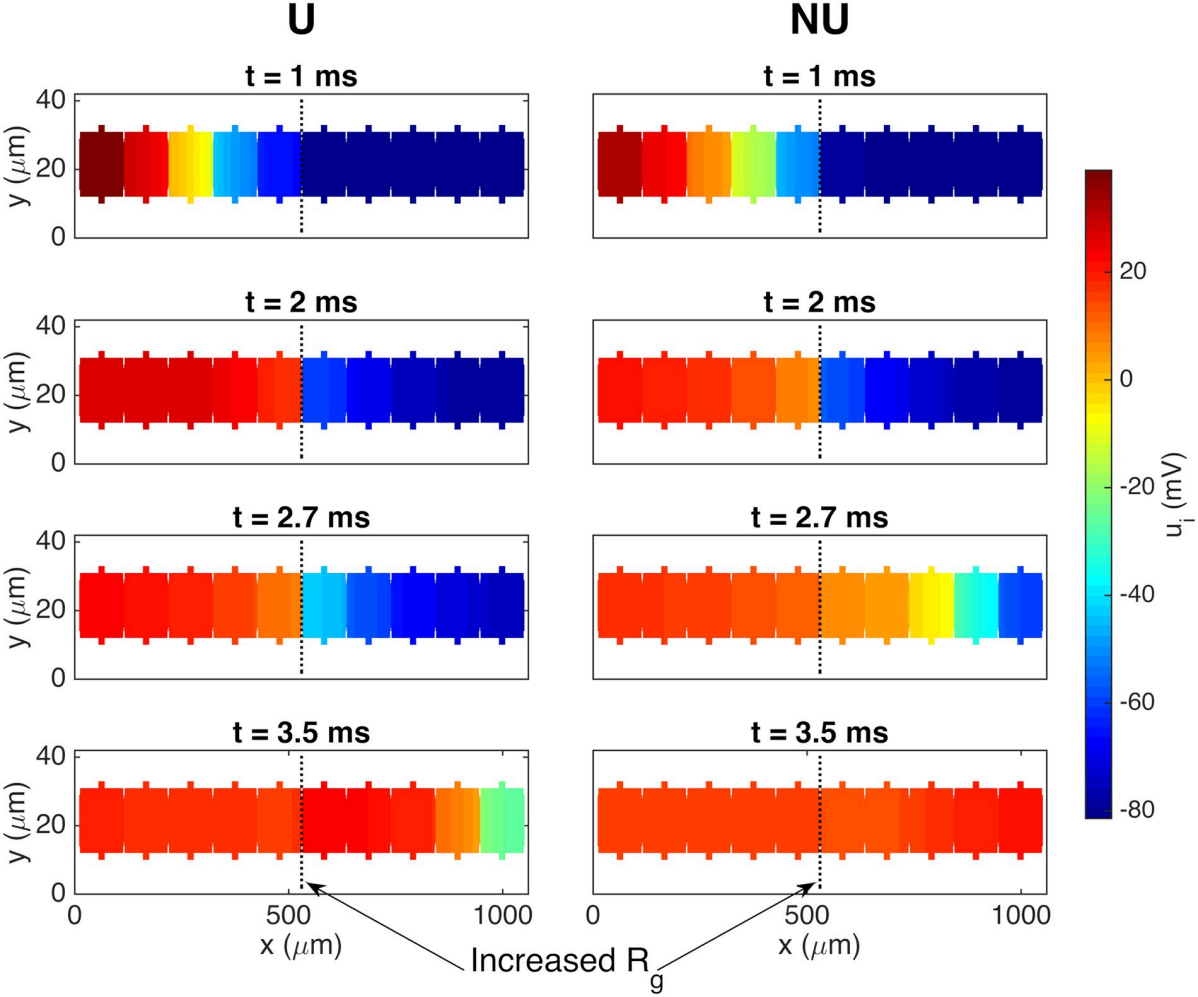
Illustration of increased gap junction delay for a uniform distribution of sodium channels compared to a non-uniform distribution. The figure shows the intracellular potential in the plane at the center of the domain in the *z*-direction at four points in time for the case with a uniform (U) and a non-uniform (NU) distribution of sodium channels. The default gap junction resistance is increased by a factor of 10 from 0.0045 kΩcm^2^ to 0.045 kΩcm^2^ between the fifth and sixth cells. The remaining parameter values used in the simulations are given in Table 1, except that the time step is set to Δ*t* = 0.01 ms.

Fig 11 illustrates the propagating wave for the uniform and non-uniform cases when the gap junction resistance is increased by a factor of 10 from the default value of 0.0045 kΩcm^2^ to 0.045 kΩcm^2^. We observe that the wave is delayed by about a millisecond when it reaches the gap junctions of reduced coupling, but that it eventually crosses the gap junctions. This happens faster for the case with a non-uniform distribution of sodium channels than for the uniform case.

**Fig 12:**
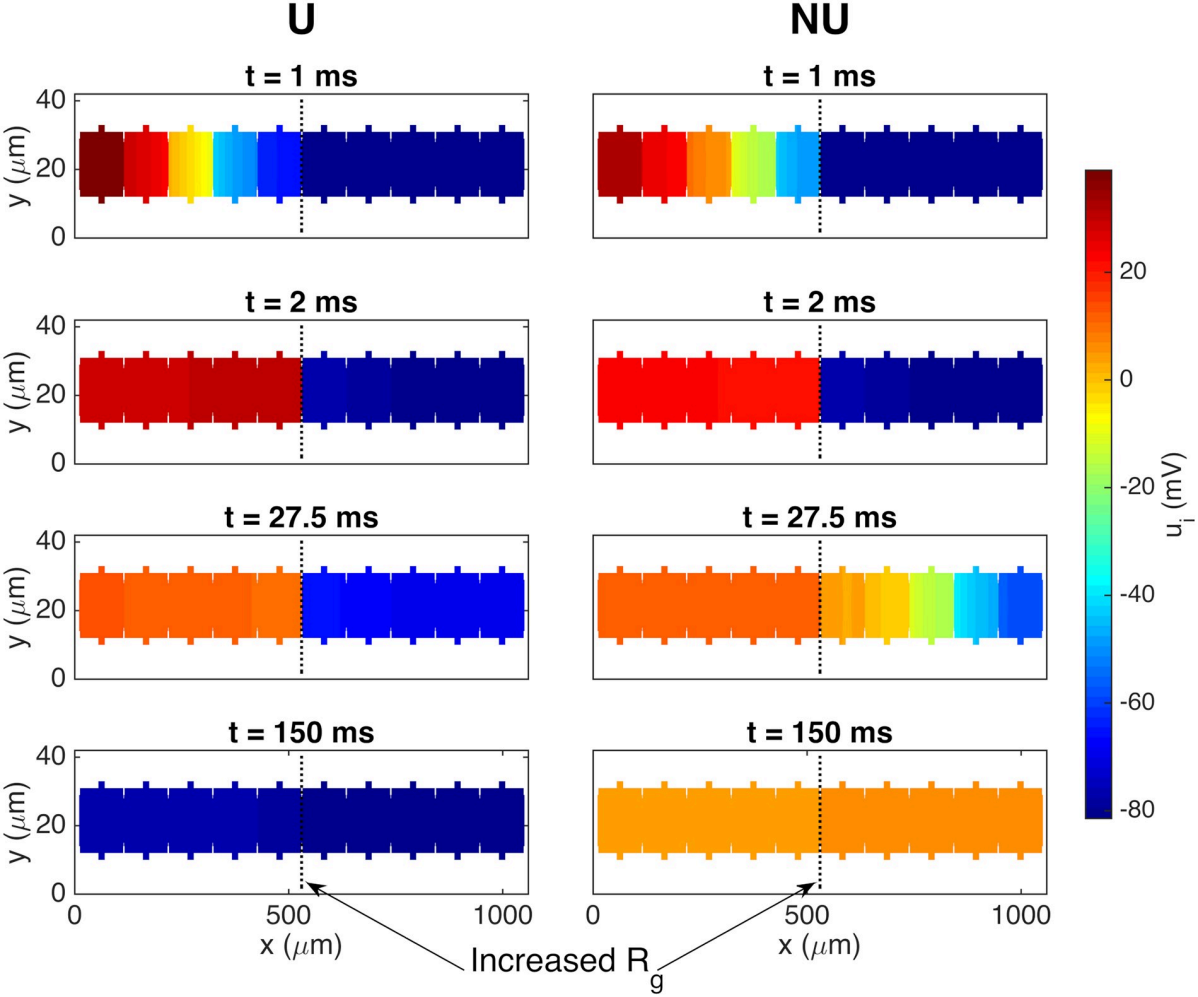
Illustration of the propagating wave being blocked for the U case but not for the NU case. The figure shows the intracellular potential in the plane at the center of the domain in the *z*-direction at four points in time for the case with a uniform (U) and a non-uniform (NU) distribution of sodium channels. The default gap junction resistance is increased by a factor of 70 from 0.0045 kΩcm^2^ to 0.315 kΩcm^2^ between the fifth and sixth cells. The remaining parameter values used in the simulations are given in Table 1, except that the time step is set to Δ*t* = 0.01 ms.

Fig 12 similarly illustrates a case in which the propagating wave is only able to cross the gap junctions of increased resistance for a non-uniform distribution of sodium channels. In this example, the gap junction resistance is increased by a factor of 70 compared to the default value on the gap junctions between the fifth and sixth cells. We observe that the propagating wave is able to cross the gap junctions of increased resistance after a long time delay for the non-uniform case, but is completely blocked in the uniform case. Also, it is worth observing that the repolarization is considerably slower in the NU case compared to the U case. However, we generally observed slower repolarization when a wave is able to propagate across a point of increased resistance, and this also holds when the sodium channels are uniformly distributed.

### Effect of cell length on the conduction velocity

In this section, we investigate how the conduction velocity depends on the cell length if the number of sodium channels per cell remains constant. Assuming that the number of sodium channels per cell remains constant, the density of sodium channels on the cell membrane will decrease as the cell length is increased. In this respect, it seems reasonable to expect that the conduction velocity would decrease if we increase the length of the cells, because the sodium channels are important for maintaining the membrane excitability necessary for cardiac conduction [57]. On the other hand, as the cell length is increased, the distance between the cell boundaries in the *x*-direction will increase, and, for a given propagation length, the propagating wave will have to cross less cell boundaries. This contrarily suggests that the conduction velocity would increase as the cell length is increased. As a result of these opposing effects, we might expect that there could be some optimal cell length which maximizes the conduction velocity.

**Fig 13:**
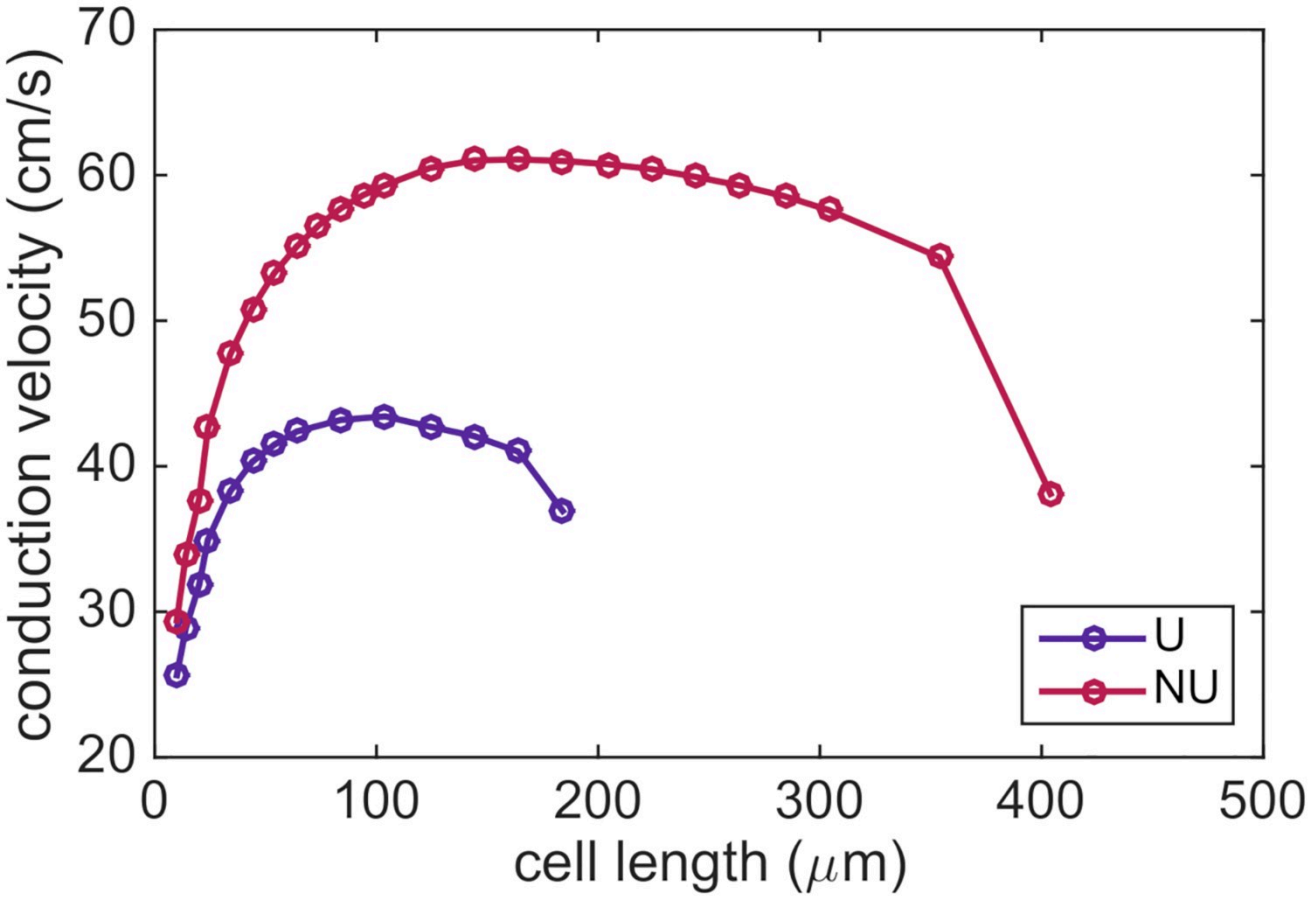
Conduction velocity as a function of cell length when the number of sodium channels per cell remains constant. The cell length here refers to the total length of Ω_O_, Ω_W_ and Ω_E_ (see Fig 2), but only the length of Ω_O_ is varied in the simulations. The remaining parameter values are given in Table 1, except that for cell lengths shorter than 20 *μ*m, the length of Ω_N_ and Ω_S_ in the *x*-direction is reduced to the cell length minus 6 *μ*m so that they fit on Ω_O_. For the simulations of cell lengths of up to 104 *μ*m, the simulation includes 20 cells, the first four cells are stimulated and the conduction velocity is calculated between cells number seven and thirteen. For the remaining cell lengths, the simulation includes ten cells, the first two cells are stimulated and the conduction velocity is computed between cells number three and seven.

In Fig 13, we investigate this property and report the conduction velocity computed for a number of simulations with different cell lengths. We consider both a uniform and a non-uniform distribution of the sodium channels. The length of Ω_O_ is varied and the cell width and the sizes of Ω_W_ and Ω_E_ are kept constant in each simulation (see the left panel of Fig 2). In order to keep the total number of sodium channels constant for each cell length, we replace the actual cell membrane area, *A*_*c*_, by the membrane area $A_{c}^{*}$ of the default cell size in Table 1 when computing the sodium channel conductance density by (22) in the NU case. In the U case, we similarly let the sodium channel conductance be scaled by a factor $A_{c}^{*}/A_{c}$.

In Fig 13, we observe that the conduction velocity indeed reaches a maximum for a given cell length and that the conduction velocity decreases as the cell length is increased or decreased from this value. In particular, for the parameters chosen here (see Table 1), a cell length of approximately 100 *μ*m and 150 *μ*m appears to lead to the maximal conduction velocity in the uniform and non-uniform cases, respectively.

### Ephaptic coupling of cardiomyocytes

Action potential propagation from cardiomyocyte to cardiomyocyte is primarily believed to be enabled by current through the gap junctions connecting individual cells [59]. However, ephaptic coupling has been proposed as an alternative or supporting mechanism for conduction between cells [19]. The EMI model is well-suited for studying this mechanism because the extracellular space is explicitly represented in the model as a geometrical subdomain.

#### Ephaptic coupling for closed gap junctions

In order to investigate the possibility of extracellular potentials alone being an alternative pathway of conduction between neighboring cells, we consider two cells with a gap junction conductance, $G_{g}\;=\;\frac{1}{R_{g}}$, set to zero on the intercalated disc between the cells. We stimulate the first half of the first cell and investigate whether the resulting propagating wave is able to pass to the second cell despite the fact that there is no current through the gap junctions between the cells.

**Fig 14:**
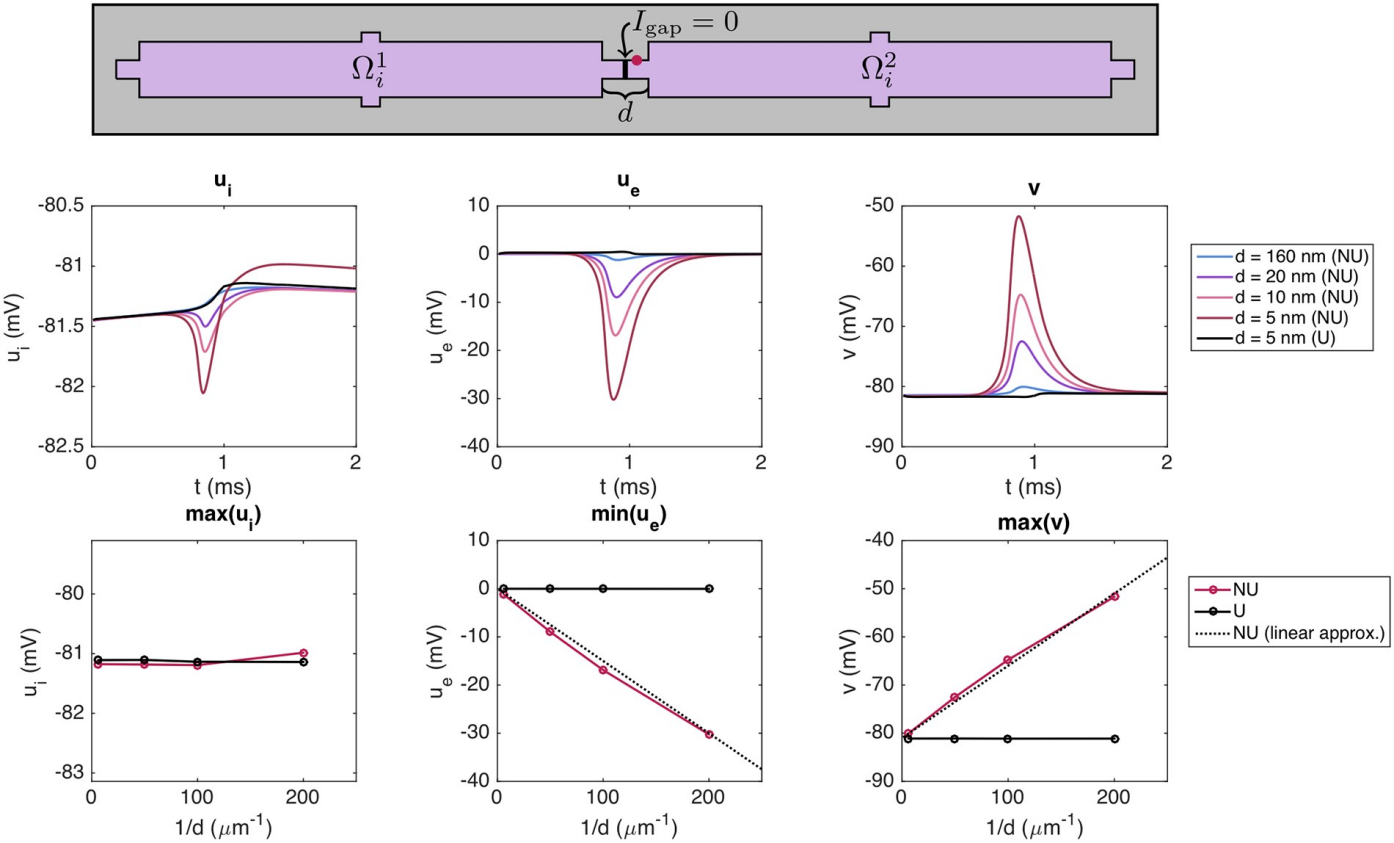
The extracellular potential between cells is highly affected by the cell distance for the NU case. The figure shows the intracellular potential, the extracellular potential and the membrane potential observed after blocked gap junctions for different values of the cell distance, *d*. The distance *d* is the combined length of Ω_W_ and Ω_E_ (see Fig 2 and the upper panel of this figure). In the NU case, we distribute all sodium channels on the vertical part of the cell ends (see Fig 3C). We consider two cells with gap junction conductance $G_{g}\;=\;\frac{1}{R_{g}}\;=\;0$ and stimulate the first half of the first cell. The potentials are recorded in a grid point located just after the blocked gap junctions, on the membrane of the second cell, illustrated by a red circle in the upper panel. The parameter values used in the simulations are given in Table 1, except that we use Δ*t* = 0.01 ms, Δ*z* = 1 *μ*m and a slightly reduced cell size. We let Ω_O_ be of size 100 *μ*m × 12 *μ*m × 12 *μ*m, Ω_W_ and Ω_E_ be of size *d*/2 × 4 *μ*m × 4 *μ*m and Ω_S_ and Ω_N_ be of size 4 *μ*m × 2 *μ*m × 4 *μ*m. Furthermore, we use Δ*x* = *d*/4. The center panel shows the intracellular potential, the extracellular potential and the membrane potential as functions of time. The lower panel shows the maximum intracellular potential, the minimum extracellular potential and the maximum membrane potential as functions of 1/*d*.

In the upper panel of Fig 14, we report the intracellular potential, the extracellular potential and the membrane potential in a grid point located on the membrane of the second cell, just after the gap junctions with zero conductance. This point is illustrated by a red circle in the domain description in the panel above the plots. We consider a number of different distances *d* between the cells, and observe that as *d* is decreased, the magnitude of the minimum extracellular potential increases considerably for the non-uniform case. Indeed, for a cell distance of *d* = 5 nm, the extracellular potential reaches a value of approximately −30 mV. For the uniform case, however, the magnitude of the extracellular potential does not increase considerably, even for a cell distance of *d* = 5 nm.

In the lower panel of Fig 14, we report the maximum intracellular potential, the minimal extracellular potential and the maximum value of the membrane potential for the same grid point as a function of 1/*d*. We observe that the minimum value of *u*_*e*_ seems to be almost proportional to 1/*d* for the NU case. In the plot, we illustrate this proportionality by comparing the computed minimal extracellular potentials for the NU case to the linear approximation min(*e*_*e*_) ≈ *a*/*d*, where *a* = −0.15 mV *μ*m.

The size of the intracellular potential does not change much for the considered values of *d*, and the increased membrane potential observed in the rightmost panel of Fig 14 is therefore entirely caused by the decrease in the extracellular potential (recall that *v* = *u*_*i*_ − *u*_*e*_). We observe that for a cell distance of *d* = 5 nm, the membrane potential just after the blocked gap junction increases to about −52 mV. This is, however, not enough to initiate an action potential in the second cell, so we do not achieve successful propagation through ephaptic coupling in this case.

#### Ephaptic coupling for a decreased extracellular conductivity

**Fig 15:**
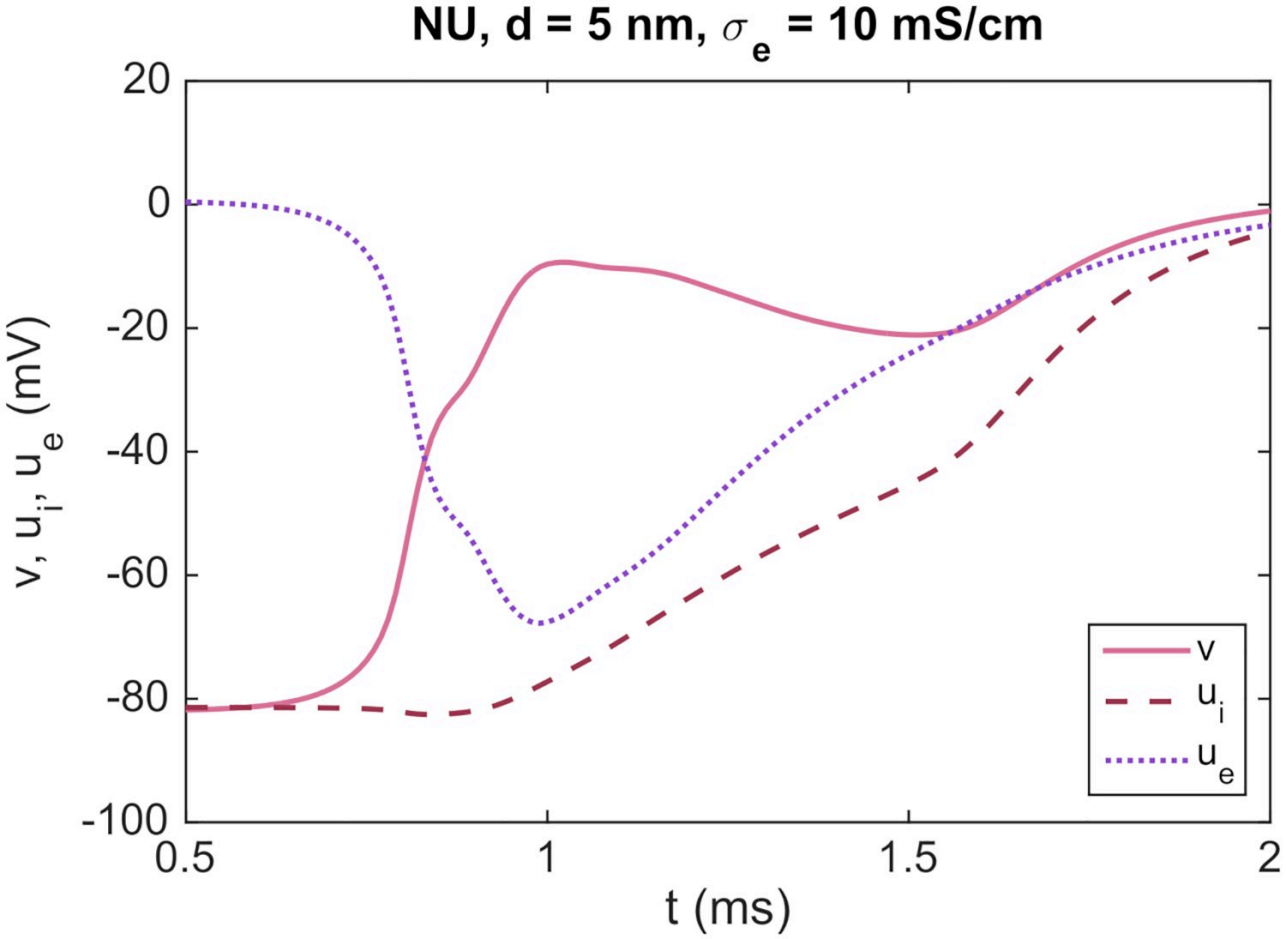
Propagation of an action potential through ephaptic coupling is achieved for a decreased extracellular conductivity. The figure shows the intracellular potential, the extracellular potential and the membrane potential observed after an intercalated disc with blocked gap junctions for a simulation with the same setup as in Fig 14, except that the value of *σ*_*e*_ is reduced from 20 mS/cm to 10 mS/cm. We consider a cell distance of *d* = 5 nm and a non-uniform distribution of sodium channels.

As observed in Fig 14, the extracellular potential reaches a value of almost −30 mV for a cell distance of 5 nm, but this is not enough to support propagation of the action potential for the case of closed gap junctions. However, this conclusion is expected to depend on the choice of parameter values used in the simulation. For example, if we assume that the extracellular conductivity is 10 mS/cm instead of the default value of 20 mS/cm, the magnitude of the extracellular potential is large enough to enable propagation though ephaptic coupling alone, as illustrated in Fig 15. Here, we show the intracellular potential, the extracellular potential and the membrane potential in the point of the membrane of the second cell illustrated by a red circle in the upper panel of Fig 14. In this case, the magnitude of the extracellular potential seems to be large enough to bring the membrane potential up to a value that triggers the activation of the sodium channels on the membrane of the second cell, and thereby to a significantly increased intracellular potential in the second cell.

#### Ephaptic effects on the *I*_Na_ dynamics

**Fig 16:**
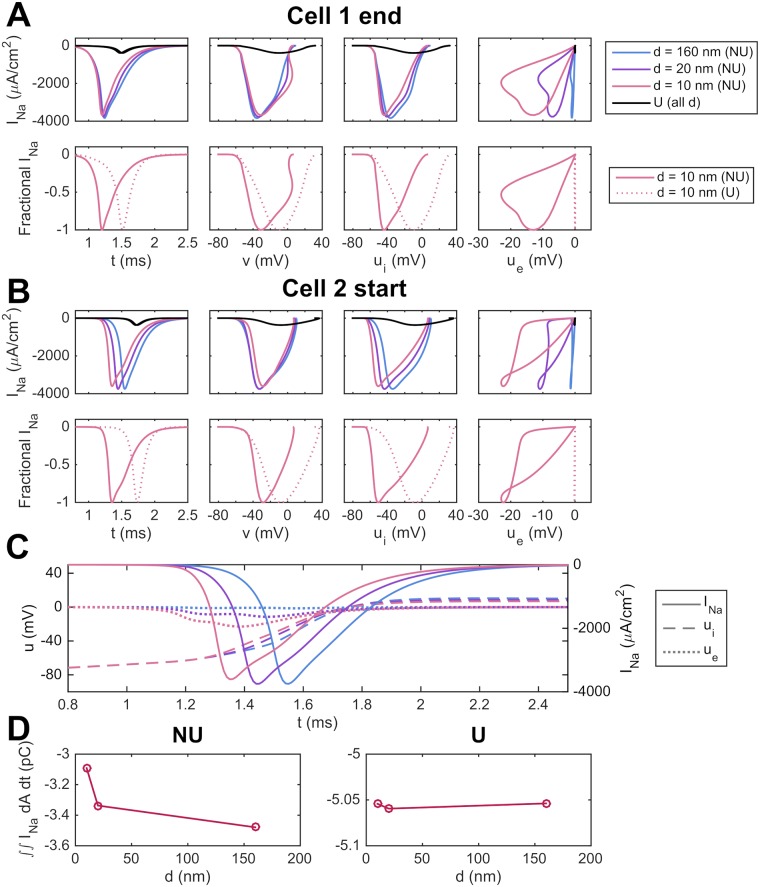
The activation dynamics of *I*_Na_ are affected by the sodium channel distribution and cell distance. (A) Raw (top panels) and normalized (fraction of peak, bottom panels) *I*_Na_ currents for the distal cell-end membrane in the first cell of a two-cell strand. *I*_Na_ in this membrane region is shown at each of three different cell distances (*d* = 5 nm, 10 nm, 160 nm) for the U and NU cases. (B) Similar results for the membrane at the beginning of the second cell. (C) Time development of *I*_Na_, *u*_*i*_ and *u*_*e*_ for the beginning of the second cell. (D) Integrated whole-cell *I*_Na_ influx in the second cell over the entire upstroke for the NU and U cases. The simulation set up and parameters are the same as in Fig 14, except that the gap junction resistance is set to the default value of Table 1.

In Fig 16, we investigate how ephaptic coupling affects the conduction properties when there is intracellular current through the gap junctions. We consider a case with two cells like in Fig 14, but where the gap junction resistance between the two cells is set to the default value given in Table 1. In particular, we investigate how the distribution of sodium channels and the cell distance affect the dynamics of the sodium channels. The figure shows that, both at the end of the first cell (A) and the start of the second cell (B), NU channel localization accelerates the rate of *I*_Na_ activation with respect to time, *v*, and both *u*_*i*_ and *u*_*e*_. These effects are exaggerated at short cell distances, particularly in the second cell (B), to which the AP is being transmitted. In panel C, the ephaptic effects on the sodium channels in the NU case are illustrated further, by considering *I*_Na_, *u*_*i*_ and *u*_*e*_ at the beginning of the second cell as functions of time. We observe that as the cell distance is decreased, there is a significant increase in the magnitude of the extracellular potential, activating the sodium channels at an earlier point in time and for a lower intracellular potential. Moreover, in panel D, we integrate the total *I*_Na_ influx over the entire membrane of the second cell. We observe that the charge movement required for the AP upstroke is reduced substantially for the NU case, especially at short cell distances. Together, these results suggest that NU localization and short cells distances may interact to potentiate sodium channel activation during the AP upstroke, thus reducing both gap junctional delay and the net charge movement required for AP propagation.

#### Ephaptic effects for large cell distances

In Figs 14–16, we observed that for small cell distances, the magnitude of the extracellular potential increases considerably in the small extracellular junctions between the cells for a non-uniform distribution of sodium channels, enabling ephaptic effects between the cells. In the remaining simulations of this paper, however, the cell distance is much larger than in Figs 14–16, and we therefore expect that the results in Figs 4–13 are not caused by ephaptic effects between the cells.

**Fig 17:**
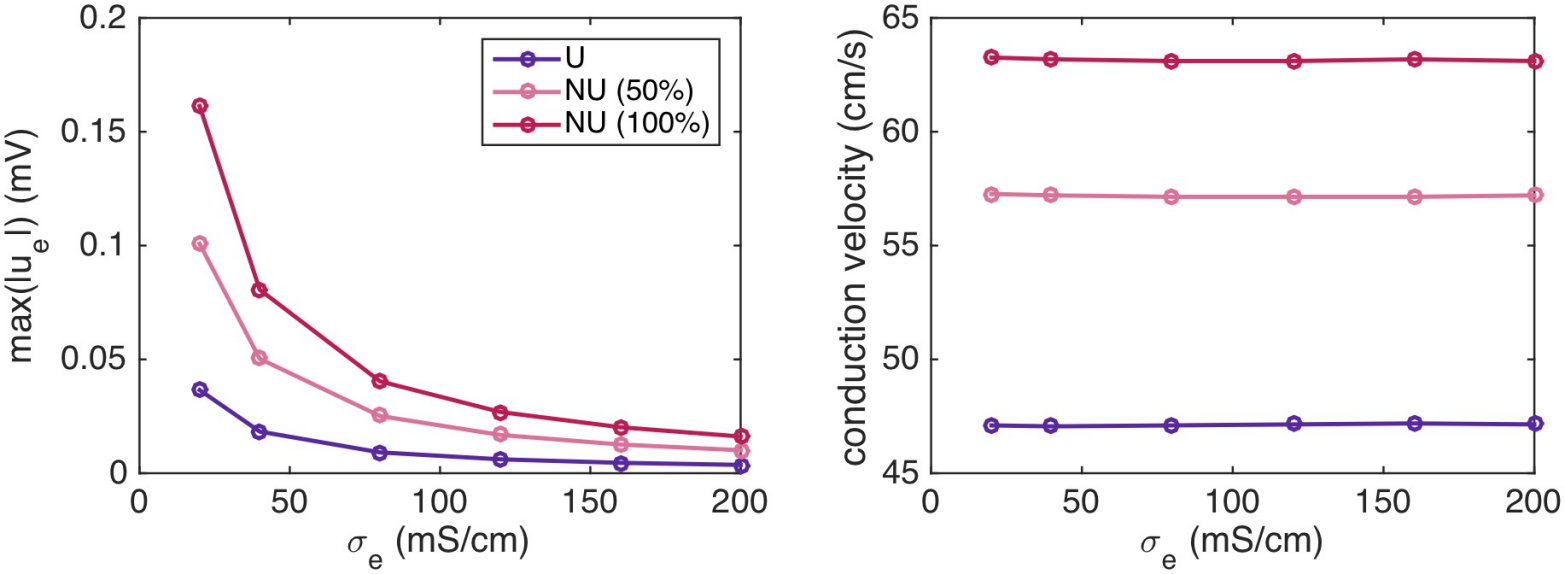
The conduction velocity for a cell distance of 4 *μ*m is nearly constant as the magnitude of the extracellular potential decreases. The left panel shows the maximum absolute value of the extracellular potential as the value of *σ*_*e*_ is increased in simulations using the same setup as in Fig 4, with 0% (U), 50% and 100% of the sodium channels moved to the cell ends. The right panel shows the conduction velocity computed in each of the simulations.

For example, Fig 17 shows the conduction velocity for 0%, 50% and 100% of the sodium channels moved to the cell ends for an increasing value of *σ*_*e*_. The left panel shows the maximum absolute value of *u*_*e*_ during the simulation for each of these values of *σ*_*e*_, and the right panel shows the corresponding conduction velocities. We observe that as the value of *σ*_*e*_ increases, the magnitude of the extracellular potential decreases, but the conduction velocity seems to remain constant. In other words, the effect that the conduction velocity is increased for a non-uniform distribution of sodium channels seems to be unaffected by a decreased magnitude of the extracellular potential, and the results of Fig 4 therefore do not seem to be caused by ephaptic effects.

## Discussion

In this paper, we have illustrated how the so-called EMI model may be used to study properties of cardiac conduction. In this model, the cardiac tissue is separated into individual cells connected to each other by gap junctions and to the surrounding extracellular space by a cell membrane, all represented as geometrical parts of the domain. As described above, this explicit representation of the membrane, the intercalated discs and the intracellular and extracellular spaces makes the EMI model suitable for studying properties that are not conveniently studied using the classical homogenized models commonly used to study cardiac conduction (see e.g., [34]). On the other hand, the increased detail of the EMI model is associated with increased computational demands, which impose limitations on the simulations currently achievable [39]. In this section, we discuss the results obtained in this study and the choice of models and parameters used in our investigations.

### Alternative discontinuous tissue models

In addition to the EMI model, several other modelling frameworks have been introduced as alternatives to the homogenized models of cardiac tissue (e.g., [40, 41, 22, 25, 26, 27, 23]). These models all represent the discrete nature of cardiac tissue with different levels of complexity, and most of the models rely on simplifying assumptions that may lead to significantly lower computational demands than the full EMI model.

#### 1D models of a single strand of cells

Some of the simplest models used to study the discontinuous nature of cardiac propagation are based on circuit diagrams of the currents along a 1D strand of cells (see e.g., [57, 60, 22, 21, 20]). In these models, each cell is discretized into a number of nodes in the *x*-direction and the cell is assumed to be isopotential in the *y*- and *z*-directions. In addition, the extracellular potential is usually assumed to be constant outside the main part of the cell, but allowed to vary in the small junctional clefts between the cells for models investigating ephaptic coupling. The gap junctions are usually represented as resistive pathways between the cells, and the 1D model is derived by applying Kirchhoff’s current law in each of the computational nodes along the cell strand.

Simulations of these models have found that a non-uniform distribution of sodium channels affects the conduction velocity and that conduction of electrical signals from one cell to the next is possible without gap junctional coupling [20, 22]. Because of the simplicity of the model, mathematical considerations regarding the parameters necessary for successful ephaptic coupling have also been conducted [21].

#### 2D sheet models

The discontinuous nature of cardiac tissue has also been represented using 2D tissue models consisting of a single sheet of cells with explicit representations of the cell boundaries and discrete representations of the gap junctions (see e.g., [61, 40, 41, 13, 62, 63, 64, 65, 66]). Some of these studies assume a negligible effect of the extracellular potential [61, 40, 13, 62, 63], while others introduce a 2D layer of extracellular space above the intracellular 2D sheet [41]. In addition, a monodomain reduction has been applied to the modeling framework, incorporating the extracellular resistivity in the equation for the membrane potential [64, 65, 66].

The 2D sheet models have been extensively used to study the effect of the distribution of gap junctions, the cell geometry and the tissue structure. For example, simulations have shown that local changes in the conduction properties may change the propagating wave front over large tissue areas [61] and that reentrant activity in the heart may be initiated by the formation of isolated sites of wavefront breakthrough caused by microstructural variations in the cardiac tissue [65, 66]. In addition, it has been found that both the cell size, the distribution of gap junctions and the tissue structure affect the longitudinal and transverse conduction velocities [13, 62, 63].

#### 3D microdomain models

Furthermore, a 3D microdomain model has been used in studies of ephaptic coupling of cardiomyocytes [25, 26, 27]. In this model, the extracellular potential is assumed to be uniform across the shortest width between the cells. In addition, the intracellular space of each cell is either assumed to be isopotential or discretized with a much coarser resolution than what has been used in our simulations. Studies using this microdomain model have found that ephaptic effects may have a significant effect on the properties of conduction [25, 26, 27]. Moreover, it was found that the ephaptic effects are not restricted to the junctional clefts between cells, but occur in all areas of the tissue with small extracellular spaces.

Because of the simplified representation of the intracellular and extracellular domains, the microdomain model is clearly more computationally efficient than the full EMI model. Thus, the model allows for simulations of, for example, smaller cell distances and larger collections of cells than what we have currently been able to handle computationally using the EMI model.

#### Models including diffusion of ions

From another point of view, the EMI model formulated in (1)–(10) is also a simplified representation of the electrophysiological properties of cardiac tissue, and more details could have been included at the cost of even larger computational demands. For example, the model ignores the effect of diffusion of ions, which could have an effect on the properties of conduction. The intracellular diffusion of calcium, for instance, may influence the conduction properties (see e.g., [2]). In our computations, we use a very simplified representation of the intracellular ion dynamics and represent the ionic concentrations only as a part of the action potential model governing the membrane dynamics. In other words, we only define ionic concentrations in the nodes located on the membrane.

Furthermore, local changes in the extracellular potassium and sodium concentrations in the narrow junctional clefts separating the cells have been proposed to have significant effects on the conduction from cell to cell (see e.g., [2, 19, 22, 23, 67]). For example, accumulation of potassium in the junctional cleft has been included in a 1D strand model and it was found that including accumulation of potassium increased the conduction velocity and allowed propagation in cases where conduction was blocked in a model without potassium accumulation [67]. Moreover, a detailed 3D model including diffusion of ions has been used to study properties of conduction under reduced gap-junction coupling [23]. In this study, it was observed that ionic concentrations varied siginficantly in the narrow clefts between cells during propagation. The study also achieved cell coupling through ephaptic coupling for a non-uniform sodium channel distribution if the distance between the cells was small enough. However, the distance needed to achieve cell coupling through ephaptic coupling was smaller than what was needed in [22] using a 1D single strand model.

### Membrane dynamics

To model the membrane dynamics, *I*_ion_, we have used the Grandi et al. action potential model [55]. This model is constructed to represent the action potential of human ventricular cardiomyocytes, and a large number of alternative models of varying complexity exists (e.g., [68, 69, 70, 71]). It would be straightforward to replace our choice of membrane model by any of these models, but we expect that the qualitative behavior observed in our simulations would be quite similar for other choices of models.

### Gap junctions

We have used a simple, passive model for the ionic currents through the gap junctions between cells, *I*_gap_, in our computations. However, experimental studies have indicated that the function of the gap junctions is in fact regulated by the voltage difference between the neighboring cells, *w*. Particularly, the gap junction resistance has been shown to increase as *w* increases [72, 73, 74, 75, 76]. Several models of voltage gated gap junctions have been proposed (e.g., [77, 78, 72, 73, 79, 80]), and such a model could easily be incorporated into the EMI model by, for example, considering a gap junction model of the form

$$I_{\text{gap}}=\frac{1}{R_{g}}gw,$$ (25)

where *g* ∈ [0, 1] is a dynamic gating variable for the gap junctions (see e.g., [29]).

### Cell and tissue geometry

In our simulations, we have used a very simplified representation of the geometry of cardiac tissue with cells shaped as rectangular cuboids with smaller rectangular cuboids at the sites of cell connections (see Fig 2). This geometry is chosen for its simplicity, and a more brick like structure (e.g. like in [27, 50, 48]) would be a more realistic representation of cardiac tissue. In addition, we have considered only very small collections of cardiac cells, organized in a single strand, and the distance between the cells has been quite large (4 *μ*m) in most of the simulations. Because of computational limitations, we have not been able to represent more densely packed three-dimensional cardiac fibers, which would have been a more realistic representation of the structure of cardiac tissue (see e.g., [48]).

Furthermore, in the majority of the simulations of a non-uniform distribution of sodium channels, the sodium channels are placed at the horizontal membrane surfaces close to the cell connections (see Fig 3B). This was done in order to place to sodium channels as close as possible to the cell transitions for the cases when the cell distance was quite large (4 *μ*m). A more realistic representation would be to place the sodium channels on the vertical intercalated discs between cells with small cell distances. However, that would have resulted in intractable computational problems.

### Computational costs

As mentioned above, the representation of the intracellular and extracellular spaces as separate geometrical domains makes the computational costs of the EMI model larger than those of the classical bidomain or monodomain models, which represents the tissue in a homogenized manner, thus permitting much coarser spatial resolutions. This has limited the number of cells we have been able to include in our simulations. Note, however, that in the simulations, a large portion of the CPU time is typically spent solving the equations for the membrane dynamics [39], and for 3D simulations of the bidomain or monodomain models, the membrane is assumed to exist everywhere in the 3D volume, whereas in the EMI model, the membrane is represented only on a 2D surface. Therefore, as the number of computational nodes increases (either because of a refined discretization or because of a larger domain), the number of membrane nodes will grow faster for the bidomain or monodomain models than for the EMI model. The EMI model could therefore theoretically be more computationally effective than the bidomain and monodomain models for a very large number of computational nodes.

Note also that the computational costs of the EMI model may be reduced by for example using different spatial resolutions in different parts of the mesh (e.g. using a finer resolution in the extracellular space between cells than in the main intracellular domain) or by reducing the domain size by exploiting the geometrical symmetry of the domain (e.g. in the *z*-direction).

### Non-uniform distribution of sodium channels

It has been demonstrated experimentally in a number of studies that the sodium channels on the membrane of cardiomyocytes are highly localized at the intercalated discs between neighboring cells (see e.g., [30, 31, 32, 22, 28, 17]), but precisely how such a non-uniform distribution of sodium channels affects the properties of cardiac conduction is still considered an open question [33].

A non-uniform distribution of ion channels on the cell membrane is, as described above, hard to represent using the classical bidomain and monodomain models because in these models, the intracellular space, the extracellular space and the cell membrane are all assumed to exist everywhere in the tissue. In the EMI model, on the other hand, the membrane of each individual cell is represented as a geometrical part of the domain, and it is therefore straightforward to represent different spatial distributions of ion channels on the cell membrane.

In this paper, we have therefore used the EMI model to investigate how different properties of cardiac conduction are affected by a non-uniform distribution of sodium channels, and we found that a number of properties were highly affected by this distribution.

#### Conduction velocity increases for a non-uniform distribution of sodium channels

First, we investigated the effect of a non-uniform sodium channel distribution on the conduction velocity and found that as a larger percentage of sodium channels were moved to the cell ends, the conduction velocity increased (see Fig 4). This is the opposite effect of what was found in earlier computational studies [22, 23, 25], which found that the conduction velocity was higher for a uniform distribution of sodium channels than for a non-uniform distribution for normal values of *R*_*g*_. The different effects observed in the different studies could be due to different choices of models and parameters. In addition, the effect could be influenced by the small, more realistic, cell distances used in the previous studies (in the range 5 nm–1 *μ*m) compared to the large cell distance used in our simulations (4 *μ*m). In fact, in the microdomain study [25], the conduction velocity was slightly higher for a non-uniform distribution than for a uniform distribution if the cell distance was large. As observed in Figs 14 and 17, a large cell distance results in very limited effects on the extracellular potential in the junctional cleft between cells. This means that potential ephaptic effects on the conduction velocity might not be adequately represented in our simulations. Specifically, the ephaptic effects are believed to potentially lead to a decreased conduction velocity due to a decreased driving force on *I*_Na_ [22]. This process is termed self-attenuation of *I*_Na_. Because of the extreme resolution needed to represent cells located realistically close, we have not been able to study how such effects impact conduction velocity in our computations.

#### Discontinuous conduction

In Figs 5 and 6, we illustrated the discontinuous nature of conduction by plotting the activation times along a strand of cells and along a single cell. We observed that as the gap junction resistance was increased, and the time delays across the gap junctions were increased, the propagating wave crossed the intracellular domain faster. Related results have been found previously, showing that the maximal upstroke velocity of the membrane potential, (*dv*/*dt*)_max_ increases for moderately reduced gap junction coupling [5, 11, 57].

Moreover, we observed differences for the uniform and non-uniform distributions of sodium channels. As also observed in Fig 10, we found that the time delays across the gap junctions were increased for the uniform case compared to the non-uniform case. In Fig 6, we also observed a clear difference in the local variations of the activation curves over a single cell. Specifically, for a uniform distribution of sodium channels, the activation curve seemed to steepen towards the end of the cell, corresponding to a decrease of the local conduction velocity along the length of the cell. For a non-uniform distribution, on the other hand, the curve seemed to flatten out toward the end of the cell, corresponding to an increased local conduction velocity along the length of the cell.

#### Conduction velocity increases for non-uniform sodium channel distribution due to increased upstroke velocity and gap junction currents

We have seen that the conduction velocity increases when the sodium channels are concentrated at cell ends (see Fig 4). This may be because of reduced delay over the gap junctions or because of improved speed along each cell. By comparing the results of Fig 5 (time delay) and Fig 6 (speed along individual cells) we observe that both components contribute to increased conduction velocity for NU compared to U. However, for the parameters considered here, the effect of gap junction time delay on the conduction velocity is significantly larger than the effect of the speed within each cell.

The reduced time delay seems to be caused by significantly increased upstroke velocity and gap junction currents in the NU case (see Fig 7). The relation between time delay, upstroke velocity and gap junction current is elaborated in Fig 9 and clearly shows that increased upstroke velocity is associated with increased gap junction currents and reduced time delays between two consecutive cells.

#### Time delays across gap junctions of reduced coupling

Next, in Fig 10, we observed how the time delays across gap junctions increased as the gap junction resistance was increased. As observed earlier, there may be significant time delays across gap junctions between regions of structural inhomogeneities [60], and in our simulations, we obtained conduction delays of up to about 25 ms as the gap junction coupling was severely reduced. Moreover, we found that the time delays were consistently larger for a uniform distribution of sodium channels than for a non-uniform distribution. This is in agreement with previous computational studies [22, 23, 25, 26], which reported that the conduction velocity was larger in the non-uniform case than in the uniform case for weakly coupled cells.

### Effect of cell length on the conduction velocity

The exact effect of the cell shape and size on the conduction velocity still remains an open question (see e.g., [4]). Since the shape and size of the individual cells are explicitly represented in the EMI model, the model could be a suitable framework for studying these questions. In this study, we illustrated this capability by investigating how the conduction velocity is affected by the cell length for a constant number of sodium channels per cell, and we observed that a cell length of approximately 100 *μ*m and 150 *μ*m seemed to give an optimal conduction velocity for a uniform and non-uniform distribution of sodium channels, respectively (see Fig 13). The existence of such an optimal cell length might be due to two conflicting effects as the cell length is increased. On the one hand, an increased cell length reduces the frequency of cell boundaries, potentially leading to an increased conduction velocity. On the other hand, an increased cell length reduces the cell’s sodium channel density, potentially decreasing the conduction velocity.

### Ephaptic coupling

Ephaptic coupling between cells has long been hypothesized to have significant effects on the properties of conduction (see e.g., [81]). In particular, ephaptic coupling has been proposed as an alternative to gap junction coupling between cells [19]. However, whether ephaptic coupling contributes significantly to cardiac propagation and the potential nature of this contribution still remain open questions (see e.g., [2, 82]).

Experimental studies supporting the hypothesis of cell coupling through ephaptic coupling includes studies of mice and guinea pigs with down-regulated expression of Cx43, which is the most abundant gap junction protein found in mammalian ventricular cardiomyocytes. The results of these studies are contradictory, with some studies showing a 17–44% reduction of ventricular conduction velocity for approximately 50% reduction of Cx43 [83, 84, 85, 86], while others found no decrease in conduction velocity [87, 88, 89, 90, 91]. It has been proposed that the difference in these studies might be explained by different extracellular conditions (see e.g., [26]), thus supporting the significance of ephaptic effects. In addition, successful propagation was observed for cell strands with no Cx43 present, although this propagation was very slow and highly discontinuous [90]. Moreover, it has been shown experimentally that the conduction velocity (especially in the transverse direction) decreased as the intercellular distance was increased [17]. This is the opposite of what is expected by classical cable theory, ignoring ephaptic effects, and the result therefore supports the significance of ephaptic effects on conduction. On the other hand, as reported in [2], the mannitol used to increase the extracellular volume, simultaneously reduces the cell width, and it is hard to differentiate between the effects of these individual geometrical changes.

#### Ephaptic coupling as an alternative to gap junction coupling

A large number of computational studies have been conducted, investigating the effect of ephaptic coupling using mathematical models of varying detail and complexity (e.g., [21, 22, 23, 24, 25, 26, 27]). These studies have indicated that ephaptic coupling could potentially serve as an alternative to gap junction coupling, but most studies found that this effect relies on a non-uniform distribution of sodium channels and a small distance between the cells. Moreover, the exact cell distance needed to obtain propagation through ephaptic coupling alone varies for the different models used in the studies. For example, Kucera et al. [22] used a 1D strand model and modelled the ionic currents by a version of the Luo-Rudy ventricular action potential model [92]. In their study, propagation by ephaptic coupling was achieved for a cell distance of 35 nm. Mori et al. [23], on the other hand, needed a cell distance of 5 nm to achieve propagation through ephaptic coupling. Their study used a model more similar to the EMI model, but with the effects of ionic diffusion included. The ionic currents over the membrane were modelled by a modified version of the Bondarenko et al. model [93] for the action potential of mouse ventricular cardiomyocytes.

In our simulations, we investigated the possibility of conduction through ephaptic coupling by considering two cardiac cells with no conductance through the gap junctions between them. In Fig 14, we observed that as the distance between the cells was decreased, the extracellular potential in the cleft between the cells decreased significantly for a non-uniform sodium channel distribution, and the minimal extracellular potential appeared to be close to inversely proportional to the cell distance. For the smallest cell distance considered in our computations (*d* = 5 nm), the extracellular potential in the cleft reached a value of approximately −30 mV, leading to a corresponding increase in the membrane potential just after the closed gap junctions. This effect seems to support the concept of cell coupling through ephaptic coupling. However, the increased membrane potential was not enough to trigger an action potential in the second cell, so we did not obtain successful propagation through ephaptic coupling in this case. On the other hand, this result is expected to depend on the choice of parameters used in the simulations. Indeed, in Fig 15 we observed that when the value of the extracellular conductivity was decreased from 20 mS/cm to 10 mS/cm, the propagating wave was able to travel from one cell to the next despite the fact that the gap junction conductance was zero.

#### Ephaptic effects of the *I*_Na_ dynamics

In addition, we investigated the effect of ephaptic coupling on the sodium channel dynamics in the case of an open gap junction. These effects have recently been systematically explored in a detailed 2D model of the intercalated disc in a voltage clamp configuration [94]. In the simulations of [94], two types of ephaptic effects were observed—self-activation and self-attenuation. At intracellular potentials far above the *I*_Na_ activation threshold, the development of large extracellular potentials rapidly brought the membrane potential close to the sodium equilibrium potential, thereby reducing the driving force for *I*_Na_ and resulting in self-attenuation of the current (and a lower peak *I*_Na_). At potentials near the threshold, on the other hand, the large extracellular potentials were capable of accelerating the channel activation, leading to a higher peak *I*_Na_.

In our computations, we investigated the effects during an action potential upstroke using the EMI model with two connected cells. We observed that, for a non-uniform distribution of sodium channels, the sodium channels were activated faster and at a lower intracellular potential as the distance between the cells was reduced. This suggests that ephaptic effects between cells might contribute to potentiate sodium channel activation during the upstroke of the action potential. On the other hand, the peak *I*_Na_ was slightly reduced for a decreasing cell distance. In addition, we observed that the integral of *I*_Na_ was markedly smaller for the NU distribution than for the U distribution of sodium channels, indicating that the net charge movement required for action potential propagation might be reduced for the NU distribution.

#### Ephaptic effects for large cell distances

In Figs 14–16, we observed that the magnitude of the extracellular potential increased considerably in the small extracellular junctions between the cells as the cell distance was decreased for a non-uniform distribution of sodium channels. This effect was shown both to impact the dynamics of the sodium channels when the gap junctions were open (see Fig 16) and to potentially enable propagation of an action potential from one cell to the next when the gap junction conductance was zero (see Fig 15).

However, in the remaining simulations of this paper the cell distance was much larger than in Figs 14–16, and the magnitude of the extracellular potential and the resulting ephaptic effects are therefore expected to be much smaller in these cases. Indeed, in Fig 17, we observed that the magnitude of the extracellular potential in the simulations measuring the conduction velocity (Fig 4) was much smaller than for the small cell distances of Figs 14–16. Moreover, the observation that a non-uniform distribution of sodium channels resulted in an increased conduction velocity seemed to be unaffected as the extracellular conductivity, *σ*_*e*_, was increased, leading to a smaller magnitude of the extracellular potential. We therefore conclude that the increased conduction velocity observed for a non-uniform distribution of sodium channels in Fig 4 is not caused by ephaptic effects, but rather by decreased conduction delays over the gap junctions caused by an increased upstroke velocity and increased gap junction currents resulting from the relocation of sodium channels to a location close to the cell ends (see Fig 7).

On the other hand, this result might have been influenced by ephaptic effects if the cell distance in the simulations had been smaller. For example, the fact that the sodium channels were activated faster as the cells distance was decreased in Fig 16 suggests that the conduction velocity might be even higher for a non-uniform distribution of sodium channels in simulations with smaller cell distances. Conversely, the fact that the peak sodium current was slightly reduced as the cell distance was decreased could potentially lead to a lower conduction velocity for cells placed close enough to exhibit ephaptic effects. In addition, ephaptic effects could be expected to lead to even shorter time delays over gap junctions of reduced coupling for a non-uniform sodium channel distribution than that observed in Fig 10. Because of computational challenges, we have not been able to study the potential ephaptic effects on the results of Figs 4–13, but these effects may be investigated in future studies using more efficient numerical strategies for the EMI model, enabling larger cell collections with small cell distances.

### Conclusion

In this paper we have used a detailed mathematical model to investigate the properties of electrical conduction in small collections of cardiomyocytes. We have compared uniform (U) and non-uniform (NU) distributions of sodium channels and found significant differences. In particular, the conduction velocity is higher and the conduction delays over gap junctions are shorter when the NU case is compared to the U case. In addition, we have illustrated differences between the optimal cell lengths with respect to conduction velocity for the two cases and seen that for the NU case, the magnitude of the extracellular potential between cells increases considerably as the cell distance is decreased.
